# Recent Advances and Applications of Bacterial Cellulose in Biomedicine

**DOI:** 10.3390/polym13030412

**Published:** 2021-01-28

**Authors:** Sam Swingler, Abhishek Gupta, Hazel Gibson, Marek Kowalczuk, Wayne Heaselgrave, Iza Radecka

**Affiliations:** 1 Wolverhampton School of Sciences, Faculty of Science and Engineering, University of Wolverhampton, Wulfruna Street, Wolverhampton WV1 1LY, UK; h.gibson@wlv.ac.uk; 2 Research Institute in Healthcare Science, Faculty of Science and Engineering, University of Wolverhampton, Wulfruna Street, Wolverhampton WV1 1LY, UK; a.gupta@wlv.ac.uk (A.G.); w.heaselgrave@wlv.ac.uk (W.H.); 3 School of Pharmacy, Faculty of Science and Engineering, University of Wolverhampton, Wulfruna Street, Wolverhampton WV1 1LY, UK; 4 Centre of Polymer and Carbon Materials, Polish Academy of Sciences, M. Curie-Sklodowskiej 34, 41-819 Zabrze, Poland; marek.kowalczuk@cmpw-pan.edu.pl; 5 Department of Biomedical Science, University of Wolverhampton, MA Building, Wulfruna Street, Wolverhampton WV1 1LY, UK

**Keywords:** bacterial cellulose, biopolymer, hydrogel, wound dressing, drug carrier, biomedicine

## Abstract

Bacterial cellulose (BC) is an extracellular polymer produced by *Komagateibacter xylinus,* which has been shown to possess a multitude of properties, which makes it innately useful as a next-generation biopolymer. The structure of BC is comprised of glucose monomer units polymerised by cellulose synthase in β-1-4 glucan chains which form uniaxially orientated BC fibril bundles which measure 3–8 nm in diameter. BC is chemically identical to vegetal cellulose. However, when BC is compared with other natural or synthetic analogues, it shows a much higher performance in biomedical applications, potable treatment, nano-filters and functional applications. The main reason for this superiority is due to the high level of chemical purity, nano-fibrillar matrix and crystallinity. Upon using BC as a carrier or scaffold with other materials, unique and novel characteristics can be observed, which are all relatable to the features of BC. These properties, which include high tensile strength, high water holding capabilities and microfibrillar matrices, coupled with the overall physicochemical assets of bacterial cellulose makes it an ideal candidate for further scientific research into biopolymer development. This review thoroughly explores several areas in which BC is being investigated, ranging from biomedical applications to electronic applications, with a focus on the use as a next-generation wound dressing. The purpose of this review is to consolidate and discuss the most recent advancements in the applications of bacterial cellulose, primarily in biomedicine, but also in biotechnology.

## 1. Introduction

One of the most abundant polymeric materials that can be found in nature is cellulose, being found in all plant life, and produced by several microbial organisms. Overall, the primary source of cellulose, which is exploited, comes from wood in a vegetal form, as this is currently the only reliable source that can keep up with the industrial demand for cellulose [1].

In addition to trees, various plants, such as flax, cotton and hemp, also contain a large quantity of cellulose. These sources are exploited to produce specific materials, including currency, specialist paper and clothing [2]. Moreover, the production of cellulose can also be obtained from fungi, seaweed and some bacterial species but, most notably, the non-pathogenic, strictly aerobic, Gram-negative bacterium *Komagateibacter* sp. (formerly *Acetobacter* and *Gluconacetobacter*), with the most studied species being *Komagateibacter xylinus (K. xylinus)* [3].

Bacterial cellulose demonstrates characteristics that are congruent in combining macromolecular and surface properties, which are crucial for in vivo and in vitro biomedical applications. Subsequently, bacterial cellulose has been shown to be a prominent novel biomaterial for biomedical exploitation. Therefore, the aim of this review is to recapitulate and discuss the most recent advances and applications of bacterial cellulose in a biomedical setting, using the bioengineered material, bacterial cellulose.

Bacterial cellulose—which increases cellular adhesion, promotes the proliferation, migration and eventual differentiation of cells, thereby increasing the rate of re-epithelialisation and leading to faster healing wounds—stands at the foreground of regenerative medicine [4]. Biomedical devices made from bacterial cellulose exhibit key characteristics, such as low toxicity, and have the ability to maintain a moist wound environment, allowing for sufficient gaseous exchange, the absorption of blood and exudate, low tissue adherence and thermal insulation. Due to the need for all these conditions to be met to produce a superior biomaterial, the design and manufacture of the bacterial cellulose needs to have a sufficiently interactive surface to allow for cell contact [5]. The arbitrated interfacial connection between cells and the bacterial cellulose plays a critical role in the performance of in vivo biomaterials, which have been developed for implants, drug delivery systems, wound healing, scaffolds and tissue/organ regeneration. The overall outcome of novel biomedical devices is determined by the way in which cells respond to the biomaterials; these interactions are usually related to physicochemical and pharmacological properties, including surface charge, wettability, topography and the presence of hydrophobic/hydrophilic compounds. These properties also influence the intrinsically biocompatible system and mechanical qualities of the material.

The following review aims at combining the most current wealth of information pertaining to modifications and applications of bacterial cellulose to be used in biotechnological and biomedical applications. Biotechnological areas that have been explored include: drug delivery systems, tissue regeneration and surgical materials, immobilisation matrices and electronics, while biomedical areas focus on the application of bacterial cellulose in wound dressings.

## 2. Production of Bacterial Cellulose

**Figure 1:**
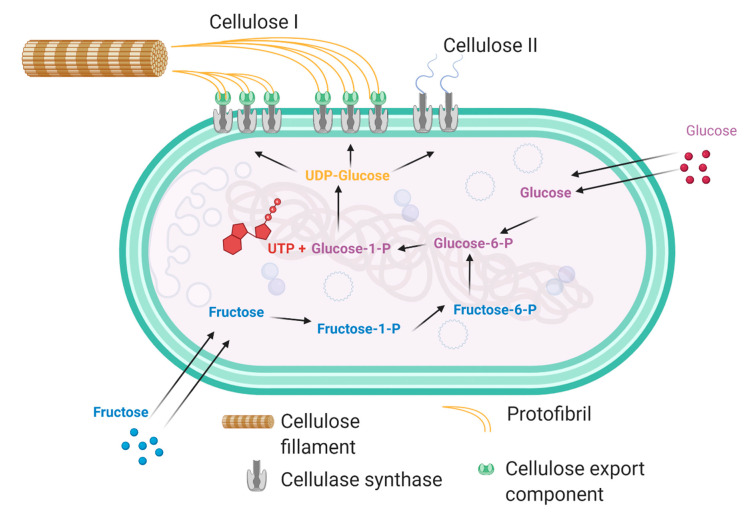
Schematic diagram of the biosynthesis of bacterial cellulose I and II from glucose and fructose.

Through the fermentation of sugars, *K. xylinus* produces bacterial cellulose extracellularly in four allomorphic forms, I–IV; however, celluloses I and II are the most widely studied, as seen in Figure 1. It is not fully understood why these bacteria produce cellulose in such a way, but it is widely accepted that the extracellular cellulose, which forms a pellicle at the top of the growth media, allows for sufficient oxygenation to be maintained for the growth of colonies near the surface, and to prevent the bacterial colony from drying out [6]. The biosynthesis of cellulose begins with the bacteria passively absorbing glucose from the environment, which is then isomerized from glucose-6-phosphate into glucose-1-phosphate. This isomer then reacts with uridine-5-triphosphate (UTP), which forms uridine diphosphate glucose, UDP-glucose. This UDP-glucose is then catalysed by cellulose synthase A into linear 1,4 glucan chains, which are activated by cyclic-di-GMP. These cellulose chains are then excreted through pores in the bacterial cell wall. However, if the bacterium becomes starved of a glucose source, the fructose pathway will be employed, utilising the relevant enzymatic processes [7]. The biochemical pathways are modulated by a specific operon, called bacterial cellulose synthesis ABCD (bcsABCD), which was identified in *K. xylinus* in 1999 [8]. The first gene of the bcsABCD operon, bcsA, is responsible for the encoding of the catalytic subunit of the enzyme, cellulose synthase. The second gene, bcsB, is responsible for the production of a regulatory subunit found on cellulose synthase, which binds to c-di-GMP, and this is particularly crucial as this interaction triggers cellulose production. The functions of bcsC and bcsD are not yet fully understood; however, it has been speculated that bcsC has a role in the production of pores in the cell membrane and the proteins it encodes for are similar to pore-forming proteins [9,10].

Following successful fermentation, the resulting pellicle contains only cellulose, secondary metabolites, microbial biomass and remaining nutrients from the growth media, which can be easily removed to obtain a highly pure crystalline cellulose matrix. Although the molecular formula of bacterial cellulose and vegetal cellulose is highly similar, the overall physical and chemical properties of each form of cellulose differ significantly [10]. The process by which cellulose is produced in bacteria follows a strict flow of biochemical pathways as seen in Figure 1.

Most bacterial cellulose is produced via the conventional static fermentation technique, whereby *K. xylinus* can grow in shallow containers of semi-defined growth medium in a static incubator at 30 °C for 7 to 14 days, after which a thick pellicle of cellulose forms at the liquid surface interface and is easily harvested [11]. Although this is the most widely used method of producing BC, there are several limitations to its use—namely cultivation time and cost. Additionally, as the organisms are subjected to differing growth conditions, i.e., some are exposed to oxygen whilst others are subjected to anaerobic conditions, and differences in carbon source gradients can cause the production of cellulose to be uneven.

## 3. Intrinsic Properties of Bacterial Cellulose

### 3.1. Structural Properties

Cellulose is comprised of linear homopolysaccharides conjugated by β-d-glucose units linked by 1,4-β-glycosidic linkages [12]. This is because of the unique 3D structure in bacterial cellulose, which accounts for the superior physical properties in comparison to vegetal cellulose. For example, bacterial cellulose fibrils form extended aggregate networks with an average diameter of 1.5 nm, this allows for a much higher surface area, flexibility, elasticity, and tensile strength [13].

After successfully producing cellulose exopolysaccharides, the linear chains of cellulose are then organised into 10–15 polymer chains resulting in nanofibers which are then successively arranged to form microfibrils which are 100 times smaller than the vegetal counterpart. The microfibrils are then bundled together to form microfibril bundles. The grouping of the microfibril bundles forms ribbons of cellulose which are 3–4 nm thick and 70–80 nm wide. These ribbons then randomly intwine with each other to form a dense matrix of cellulose fibres that are established through heavy inter- and intra-chemical bonding; primarily hydrogen bonding, which occurs between successive sheets of cellulose. This high level of hydrogen bonding also allows for cavities to form within the cellulose, which possesses an ionic charge that allows for materials to be embedded into the material, such as silver ions. This property of cellulose will allow for manipulations to be made in terms of loading the cellulose with specific antimicrobial agents that possess an ionic charge which can target the causative organisms responsible for infections [14].

**Figure 2:**
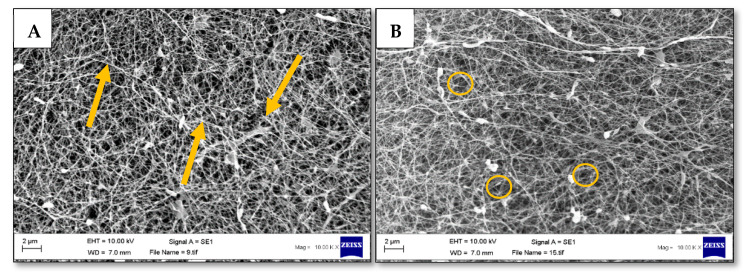
(**A,B**) Scanning electron micrographs of purified bacterial cellulose where the cellulose fibrils (arrows) and voids (circled) are clearly seen.

Bacterial cellulose Figure 2A,B is liquid and gas permeable, non-pyrogenic and hydrophilic, which make it innately suitable for biomedical applications [15]. The hydrophilicity and relatively high swelling ratio of bacterial cellulose allow it to maintain a moist environment at the wound site, while retaining an ideal temperature range of 25.3–37.3 °C [16]. The hydrophilicity of bacterial cellulose also enables the reversible swelling and de-swelling of the dressing, which can be exploited in the management of wound exudate from heavily emanating wounds [16]. In addition to being biodegradable and non-toxic, one of the main advantages which bacterial cellulose has over vegetal cellulose is the high level of innate purity, enabling its direct utilisation with minimal processing. Bacterial cellulose is chemically equivalent to vegetal cellulose, but it is free of by-products, such as hemicellulose, pectin and lignin, along with other lignocellulosic compounds [17].

### 3.2. Mechanical Properties

The spatial arrangement of the cellulose fibres determines the mechanical properties that distinguish bacterial cellulose from vegetal cellulose Table 1 These differences include a much higher crystallinity ratio in bacterial cellulose of 84–89% [18]. Compared to the crystallinity ratio of plants at 60–64% [19] and with the notable deficiency of other by-products, which are naturally found in vegetal cellulose, this allows for a much simpler purification process to be carried out to produce pure bacterial cellulose [19].

The physical properties of bacterial cellulose are strongly correlated to the processing conditions under which the cellulose is tested. For example, under dry conditions, following lyophilisation to eliminate most of the water, it was typically found that the cellulose had a tensile strength of 240 mPa, an elongation at break of 5%, a maximum strain in other order of 4% and a Young’s modulus of 12 GPa, although it has been shown that a single strand of bacterial cellulose can have a Young’s modulus of 90 GPa. This is somewhat disappointing when compared to wet bacterial cellulose (99% water), as the mechanical properties indicate a tensile strength of 400 mPa, maximum strain in the order of 20%, and Young’s modulus of 130–145 GPa [20,21,22,23]. The values for Young’s modulus in wet bacterial cellulose indicate that there is a high level of elasticity within the material in comparison to the lyophilised form. Table 1 summarizes some of the key physical properties of bacterial cellulose.

**Table 1:** Comparison of parameters for wet and lyophilised bacterial cellulose expanded [21,22,23].

Parameter	Wet Cellulose	Lyophilised Cellulose
Tensile strength	400 mPa	240 mPa
Elongation break	10%	5%
Maximum strain order	20%	4%
Young’s modulus	130–145 GPa	12 GPa

### 3.3. Water Holding/Release Capacity

Quantitative parameters included water holding capacity (WHC) and water release rate (WRR). These parameters varied considerably according to the physicochemical properties and structural qualities of the bacterial cellulose being evaluated [24]. The main aspects which affected the parameters were pore size, distribution and the available surface area. In addition to this, any presence of hydrophilic compounds, which were added to the bacterial cellulose, as part of bacterial cellulose composite, also showed a marked change in the WHC and WRR values [24,25]. The WHC of bacterial cellulose ranges from 50 to 1000 times its dry weight, depending on the conditions of synthesis. In standard static fermentation synthesis, bacterial cellulose accounts for below 2% of the weight of the pellicle, with the rest being remaining growth media [24]. An explanation for such a high water content can be attributed to the fact that the bacterial cellulose is assembled extracellularly in the liquid growth medium, where copious micelles are formed which entrap large quantities of liquid; this, in turn with the high available surface area in the interstitial regions of the bacterial cellulose, allows for more water to become trapped [6]. As the counteracting effects of WHC and WRR within the cellulose structure are becoming better understood in terms of the ability for the dressing to hold and release water, the realm of modifying the structural parameters of bacterial cellulose during the biosynthesis phase or via post-synthetic modifications has become a focal point of several studies [26,27,28,29,30].

### 3.4. Pore Size and Fibre Morphology

The water holding capacity of bacterial cellulose is directly proportional to the surface area and pore volume in the matrix. A compact cellulose matrix, which presents denser fibril arrangements resulting in a reduced pore volume and surface area, has been shown to have a lower WHC; this is because the number of sites in which water can become entrapped is diminished [25].

Pore size can also be influenced by the method of fermentation itself, be it static or agitated. Revin et al., (2019) demonstrated, under static conditions for 14 days at 37 °C, that the pore sizes in the bacterial pellicle ranged from 11–164 μm, whereas under dynamic conditions, in which the fermentation vessel was continually agitated for five days, produced bacterial cellulose pellets with pore sizes ranging from 165–330 μm. This is important when deciding whether to produce BC via static or agitated conditions as these parameters influence the overall pore size, which will influence how well additional compounds are incorporated within the matrix when producing composites [26]. The sizes of the pores also influence the mechanical and physical properties of the material, where larger pores introduce more voids within the material, whereby the density of cellulose fibres are reduced. Subsequently, the reduction in density increases the overall porosity, which allows compounds/cells to be loaded more easily [31]. However, the reduction in the density of cellulose fibres detrimentally affects the tensile strength of the material. This reduction in strength of the BC may be regained through the incorporation of additional components which will occupy the voids, thus providing the cellulose matrix with additional structural support.

### 3.5. Bacterial Cellulose-Based Biocompatible System

Biocompatibility is defined as the capability of a compound or material system to be therapeutically active once it is applied to a recipient without causing a systemic or local adverse response. For a biomaterial to be considered biocompatible, it must meet strict sensitisation assays. An initial biological assay to assess suitability is to conduct a cellular metabolic assay, such as (3-(4,5-dimethylthiazol-2-yl)-2,5-diphenyltetrazolium bromide) tetrazolium reduction assay (MTT) in which the metabolic activity of cells is measured, thus providing cell viability data [32].

The MTT assay is based on NADH dependent oxidoreductases which reduce the tetrazolium MTT dye to formazan, and this reaction is directly proportional to the number of viable cells in a sample [32]. This MTT assay was used in the determination of the cytotoxicity of bacterial cellulose loaded with mesenchymal stem cells from rabbit bone marrow against human macrophages [33].

Bacterial cellulose has been earmarked as an appropriate material to create wound dressings due to the material being able to perform well under the biocompatible system testing criteria. This can be attributed to the high purity and nanofibrous structure, which allow the cells of the host to bind and proliferate, thus aiding in the wound [33].

Several aforementioned studies have been conducted in vivo and in vitro to determine the intrinsic biocompatible properties of bacterial cellulose with different morphologies, including membranes, pellicles or pellets, which were subjected to different purifications and sterilisation techniques. They have been modified in various ways using both organic and inorganic polymers and compounds, pharmaceuticals and other holistic materials with a duration that ranged from 24 h to 1 year, all to examine the biocompatibility [34].

Regarding the recently published literature, there has been an apparent increase in the exploration of using bacterial cellulose as a wound dressing with various techniques available; however, it is rare to find studies that experiment on human subjects. Calvalcanti et al., (2017) were able to show that a bacterial cellulose wound dressing, which was applied to venous leg ulcers for 120 days, did not induce any adverse effects and decreased the overall depth of the ulcer, which would indicate that the cellulose wound dressing facilitated the remodelling of the dermal layers without any toxicity relative to conventional wound dressings [34]. The result showed no toxicity and was produced with 95% of the macrophages remaining viable. Additionally, in the same study, nitric oxide was used as an indication of cytotoxicity. Nitric oxide is produced by macrophages to eliminate pathogenic bacteria and to mediate inflammation. Again, no cytotoxicity was observed as nitric oxide levels remained steady in comparison to pure bacterial cellulose [35,36].

Colourimetric assays can quickly provide valuable data regarding cytotoxicity; however, the intrinsic biocompatible properties of bacterial cellulose and bacterial cellulose composites must also be investigated using human cell lines and animal models. One such study was by Bayazidi et al., (2018), who identified that the use of chitosan in bacterial cellulose will degrade in the presence of lysosymes into oligosaccharides, which encourages re-epithelialisation and angiogenesis at the wound bed [37]. Bacterial cellulose-chitosan composites were tested on rats with hernias by sub-dermally implanting the mesh over the hernia wound site. Following the histopathological examination of the wound site and organs for adverse alterations in dermal anatomy and fluctuations of inflammatory cells, it was observed that there were no histopathological or cytotoxic alterations with no allergic reactions or irritation; however, there was a high degree of fibroplasia though the bacterial cellulose-chitosan mesh, indicating low cytotoxicity as innate cells readily colonise the mesh [38,39,40,41,42].

Ye et al., (2019) successfully modified the nano-porous matrix in bacterial cellulose to a microporous analogue. This was accomplished by using gelatin microspheres as surface alteration agents and as porogens [43]. Gelatin has also been shown to have very low cytotoxicity as it is a by-product of collagen hydrolysis. Gelatin has been used as a surface modifier as it mimics the composition of collagen, which aids in cellular adhesion to the bacterial cellulose. The cytotoxicity of the bacteria cellulose composite was conducted against the HaCaT keratinocyte model cell line in vitro [44]. It was concluded that the cells were capable of infiltrating and proliferating within the microporous structure of the modified cellulose matrix, which is essential in tissue regeneration treatments. Similarly, investigations via in vivo analysis of C57BL/6 mice, who had dorsal dermal wounds, increased re-epithelialisation with upregulated healing processes and minimal inflammatory cell responses when bacterial cellulose-gelatin wound dressings were applied [45].

## 4. Bacterial Cellulose as a Biotechnological Material

### 4.1. Drug Delivery

As is the usual case, the most common method of loading drugs in BC membranes is via immersion in the drug solution usually following lyophilisation to allow for maximum absorption of the drug [46]. The most common drugs to be incorporated into bacterial cellulose are anti-inflammatory drugs, such as ibuprofen and diclofenac, and antimicrobial drugs [47,48,49,50,51,52]. The efficiency of bacterial cellulose as a drug delivery material can be improved to provide additional properties and functions by exploiting tensile strength and water uptake to load the cellulose with antimicrobial compounds such as antibiotics [53,54].

The addition of antimicrobials, such as antibiotics, into bacterial cellulose has also been of interest by immersive techniques. Tetracycline loading was performed by submerging bacterial cellulose in the antibiotic solution for 24 h under constant agitation. The results showed an initial exponential release for the first 3 h but plateaued to a steady release for the remaining 93 h [47]. In another study, saturating bacterial cellulose loaded with fusidic acid over 24 h of submersion showed antimicrobial activity against *Staphylococcus aureus*. This composite was reported to have increased bioactivity with high levels of action against both Gram-positive and Gram-negative bacteria [48].

An amoxicillin release study from bacterial cellulose matrices showed that the overall controlling factor of drug release was the actual concertation of amoxicillin itself [42]. Moreover, release profiles of tetracycline were studied, which showed an initial burst of activity and, after 2 h, a steady release with sufficient antimicrobial effects against *Bacillus subtilis, Staphylococcus aureus, Escherichia coli* and *Candidia albicans*, showing, with further testing, that there is low cytotoxicity against HEK293 cells [55].

Antiseptic compounds, such as octenidine, were also loaded into BC via submerging and also showed low levels of cytotoxicity against keratinocytes, with a biphasic release profile in the first release surge within 8 h, followed by a steady release up to 96 h. Moreover, this study also confirmed that providing the BC-Octenidine composite was kept moist and in the dark, it was still fully viable six months later with no loss of antimicrobial action. The emulsification of cellulose with various poloxamers gave the overall effect of controlling the release profile according to the concentration and type of poloxamer [48,52].

Anti-inflammatories, such as ibuprofen and diclofenac, can cause adverse gastrointestinal symptoms in some people, which would be an indicator for the use of a transdermal delivery system [56]. Polymethanocryolyl-glycine has been composited with bacterial cellulose to form a pH sensitive scaffold for the release of diclofenac. At pH 2, the drug is kept within the membrane. However, when exposed to the skin’s natural pH of 7.4, the delivery of diclofenac is slowly released over a period of 96 h [56,57].

Usually, drug delivery systems take the form of a patch, utilising the whole bacterial cellulose pellicle [44]; however, Hoshi et al., (2018) developed a hollow spherical ball to slowly release fluorescein. The hollow spheres were formed by combining *K. xylinus* and growth media suspension, dropwise, into a mixture of silicone oils, this was then incubated at 30 °C for 14 days [58]. As the bacteria grew, the cellulose fibrils formed spherical structures around the droplet of growth media and cells, which, after purification, left a hollow void at the centre which can be used to deliver minute amounts of drug directly to the target location [58].

Compounds that have been derived from lignin, such as coniferyl alcohol, have been used as antibacterial agents and, as such, have been used as a secondary component in bacterial cellulose, which resulted in a highly potent antibacterial wound dressing that exhibited a higher rate of revascularisation of the wound site and a decrease in pain and erythema [59].

### 4.2. Surgical Material

The mechanical properties of bacteria cellulose, such as high tensile strength, elasticity and water retention rate, combined with a low level of cytotoxic effects and low inflammation induction, coupled with an appropriate composite, makes BC an ideal material for surgical implants. An example of this is the development of vascular grafts, which are produced by perforating bacterial cellulose with a needle, then drying completely. The resulting surface of the BC was highly similar to that of the porcine femoral artery, with desirable mechanical properties. The BC graft was investigated in vivo by a homolateral femoral bypass. After 1 month, blood flow, patency and CD31 positive cells were confirmed to be normal [60,61].

Ocular surgery is one of the most sensitive areas of medicine, which require highly precise and sensitive materials, and due to the intrinsic properties of BC, it has become a focus of attention in producing synthetic corneas. The sole method for treating corneal disease is by a corneal transplant; however, there is currently a shortfall in corneal organ donors. A synthetic cornea produced from a bacterial cellulose and polyvinyl alcohol is a potential substitute, which could potentially avert the need for cornea donors. It was shown that BC alone was not a sufficient material for use as a cornea, and so had to be combined with PVA [48]. The BC/PVA composite exhibited ideal properties for use as corneal stroma. The composite was not toxic against human corneal stroma cell lines with low levels of cytotoxicity. Furthermore, after 4 weeks, in vivo studies following intrastromal transplantation in rabbits showed that the corneas remained transparent with no inflammation or sensitisation and had increased revascularisation [62,63,64].

Inoue et al., (2020) addressed an increase in demand for bioabsorbable barriers in dental medicine in the treatment of periodontal diseases. The development of a bioabsorbable and biofunctionalised form of bacterial cellulose has shown promising results [65,66]. However, key challenges in the development of these dental barriers needed to be overcome, namely, the oxidisation of BC utilising sodium metaperiodate before loading the BC with an appropriate biocidal compound. In this study, chlorhexidine was used as the drug and aqueous sodium metaperiodate (NaIO_4_)_aq_ as the oxidiser. Oxidation was achieved by placing the BC into the (NaIO_4_)_aq_ for 6 h and placed in the dark. In order to modulate the release of chlorhexidine, it was encapsulated in β-cyclodextrin to form chlorhexidine:β-cyclodextrin complexes. Following antimicrobial testing, it was reported that the modified bacterial cellulose was active against both *S. aureus* and *E. coli*, as well as *C. albicans* [66].

### 4.3. Tissue Regeneration

Tissue regeneration is at the forefront of biotechnological research and bacterial cellulose has been shown to be an ideal starting material. The intracellular matrix of BC allows it to be an ideal scaffold for a multitude of cell types. Roman et al., (2019) was able to take advantage of the porosity of BC to facilitate the growth of cartilage [32]. This was achieved by seeding 1.6 × 10^5^ bovine chondrocytes in Dulbecco’s Modified Eagle Medium onto sterile BC, which was allowed to grow overnight at 37 °C. Following SEM analysis, it was shown that the chondrocytes began to migrate into the BC matrix where they showed differentiation and chondrogenic phenotypes in cartilaginous matrix secretions [67]. To encourage the seeding of the chondrocytes, laser perforation was performed to produce channels that facilitated the migration of the cells. Furthermore, in vivo studies have shown that supplementing bacterial cellulose with alginate prior to seeding with human nasoseptal chondrocytes resulted in high levels of stable chondrocyte cell growth [68].

Though bacterial cellulose has been shown to be nontoxic with various cell lines and tissues, the capability of cells to adhere to native bacterial cellulose is not ideal due to the dense structure of the fibrillar matrix, which restricts cell migration into the BC. One method for overcoming this issue is with the use of porogens, such as agarose particles or paraffin wax, to enlarge the pores of bacterial cellulose during biosynthesis [42].

The variety of cells grown in bacterial cellulose matrices include bone, muscle, neuronal and skin tissue. The use of mesenchymal stem cells is spearheading regenerative medicine by combining bacterial cellulose and hydroxyapatite, which allowed the stem cells to differentiate into stable osteoblasts within 21 days [69,70,71,72,73].

As BC has previously shown promising re-epithelialisation in tissue implantation studies, Mandour et al., (2019) built on this to develop a novel treatment for tympanic membrane (TM) perforation repair (myringoplasty) and the restoration of the eardrum, using BC patches rather than the conventional fat or *Temporalis fascia* graft [74,75]. It was found that the BC patches were successful in treating patients with TM perforation, as up to 50% of patients (n = 40) achieved successful recovery. This can be attributed to the BC membrane graft having similar characteristics to fat grafts; in that the thickness, tensile strength and Young’s modulus of the BC was found to be 10.33 ± 0.58 μm, 11.85 ± 2.43 N/m^2^ and 11.90 ± 0.48 MPa, respectively. [74] These values coincide with the requirements of, and indeed similarities to, conventional grafts, which highlight potential implementation for standardising the use of BC in myringoplasties. Post-surgical reviews showed that there was successful migration of cells from the surrounding tissues which began to recolonise the BC matrix, leading to an assumption that the overall function of the tympanic membrane may be restored.

### 4.4. Biosensors

IUPAC (International Union of Pure and Applied Chemistry) defines a biosensor as an integrated receptor-transducer device, which provides either semiquantitative or quantitative information utilising a biologically derived sensor. The sensors used in these devices can vary in nature and include antibodies, enzymes, proteins, and cells. BC biosensors have undergone a swathe of advancements, owing to the attractive intrinsic characteristics of the material, and have found uses in pollutant, humidity, gas, food safety and biomedical applications [76].

An example of a biomedical application of biosensors was described by Li et al., (2016), who developed a dopamine detector by destroying the solid BC pellicle into a liquid through ultrasound, which was then loaded with palladium (Pd) nanoparticles [77]. This BC:Pd material was further modified by the addition of Nafion and laccase, which was then loaded onto a glass carbon electrode to complete the biosensor construction. This study showed that dopamine biosensor had a high sensitivity of 5–167 µM and a low detection limit of 1.26 µM [77].

Another application is the development of fibre-optic glucose detectors by Yu et al., (2019), who conjugated cellulose, fluorescent carbon quantum dots and glucose oxidase. The biosensor could detect glucose levels as low as 28.79 nM [78].

BC biosensors offer many advantages over conventional tests in that BC sensors are biodegradable, easily transported, small and lightweight. These properties are highly advantageous in developing novel biosensors as they allow them to be transported and employed quickly and easily, providing valuable accurate data in a short period of time.

However, there are still disadvantages to the use of biosensors in which conventional analytical techniques may not be susceptible to. Primarily, the low stability of the biological active element of the sensor, in that when the element is removed from its native environment, the activity rapidly decreases—especially in enzymes. In order to maintain the activity of enzymes within biosensors, they first have to be immobilised within the BC matrix, which may marginally extend the lifespan of the biosensor.

### 4.5. Immobilisation Matrix

The immobilisation of amino acids onto the surface of bacterial cellulose was investigated with the addition of arginine composites following the oxidation of bacterial cellulose. Consequent testing revealed that the formation of arginine in the bacterial cellulose after oxidation resulted in the surface of the material becoming more textured. It is thought that this texturing of the cellulose stimulated the propagation, migration and expression of fibroblast and endothelial cell lines [44,68,72].

The ability to immobilize enzymes on a substrate paves the way for more technologically advanced biocatalysts to be developed. Biopolymers, such as alginate/bacterial cellulose composites, have been used to immobilize some of the most widely used industrial enzymes, such as lipases and laccases. Lipases and laccases are of crucial importance in the production of biofuels and bioremediation [37,73]. It was shown that the BC–alginate composite provides up to eight-fold more activity of lipases when compared to only bacterial cellulose immobilized lipases [79].

### 4.6. Filtration

The use of bacterial cellulose as a filtration material has been recently explored due to the mesh-like fibrous network. To achieve a robust filter, various compounds have been added to the bacterial cellulose in order to enhance its specificity to filtrates. One of these compounds was graphene oxide, which resulted in a filter with high mechanical strength, water stable and highly selective ionic attraction which make it a highly attractive method of filtration for the pharmaceutical industry and in water purification. In addition to graphene oxide, palladium nanoparticles have also been incorporated which have proven to be effective filters of industrial organic dyes. It was shown that the composite BC filters were able to remove 99% of methylene orange from wastewater, along with the removal of heavy metals, showing 50% removal of copper [80].

### 4.7. Electronics

When bacterial cellulose is combined with conductive compounds, such as metal nanoparticles or graphite, it naturally becomes conductive of electricity [76,78]. It is for this reason that there has been interest in developing materials from bacterial cellulose for electrical applications. One such development was made by incorporating polyaniline and silicone nanoparticles, which made a conductive, yet flexible material, which was successfully used as an anode for lithium-ion batteries [81]. Additionally, by combining cobalt ferrite into the growth media, nanotubes of highly conducting cellulose were produced, which could be utilized as nanowires in many modern electrical devices. As a result of this, biomedical electronic engineering is a specific area could exploit conductive bacterial cellulose composites. Whilst having the ability to conduct electricity, the integral properties of BC are still intact, resulting in the material remaining highly flexible and strong [82]. An example of this is demonstrated through the oxidation of polyaniline by ammonium persulfate. This reaction yielded highly conductive particles, which, when combined with BC, produced highly effective electrical capacitors with a retention of 94.3% after 1000 cycles. [83].

Lay et al., (2017) also demonstrated that, through the polymerisation of pyrrole into polypyrrole, which used iron chloride as an oxidizing agent, they were able to coat the lyophilised BC pellicle to achieve a highly conductive biomaterial, which shows promise for a new class of biosensors—namely, energy storage devices and biosensors—with the focus on thin film transistors, organic flexible supercapacitors and organic light-emitting diodes [82,83,84,85,86].

### 4.8. Other Uses

Aside from being a highly useful and versatile biomaterial, bacterial cellulose has been consumed as food stuff for thousands of years as a pure source of dietary fibre. In Asian countries, it is consumed as a dessert known as “Nata de Coco” and has been recognized by the Food and Drug Administration as GRAS (generally regarded as safe) 1992 [87]. It is also used to produce a fermented drink called Kombucha by using a bacterial cellulose pellicle colonised with bacteria and yeasts, which is known a “SCOBY”—Symbiotic Culture of Bacteria and Yeast. The SCOBY then ferments a tea solution which results in a beverage high in probiotics. Bacterial cellulose has also been used a meat-free option for vegetarians, due to its structure, as well as being used a high fibre dietary supplement [88].

Another use of BC is the stabilization of peanut butter by using bacterial cellulose nanoparticles to stabilize oil and water Pickering emulsions. It was shown that only 0.05% (*w*/*v*) of BC was needed to fully stabilize peanut butter, showing that bacterial cellulose nanoparticles can be a highly useful food grade emulsifier [89].

In addition to food applications, bacterial cellulose has also found its way into the cosmetic industry due to it high water retention and overall texture. Bacterial cellulose can be loaded with moisturizers such as *Aloe Vera* or shea butter and used as an effective moisturizing face mask or impregnated with salicylic acid/glycolic acid for wart and verruca removal [90]. Table 2 summarises the various applications, as mentioned previously.

## 5. Wound Treatment

### 5.1. Wounds

**Figure 3:**
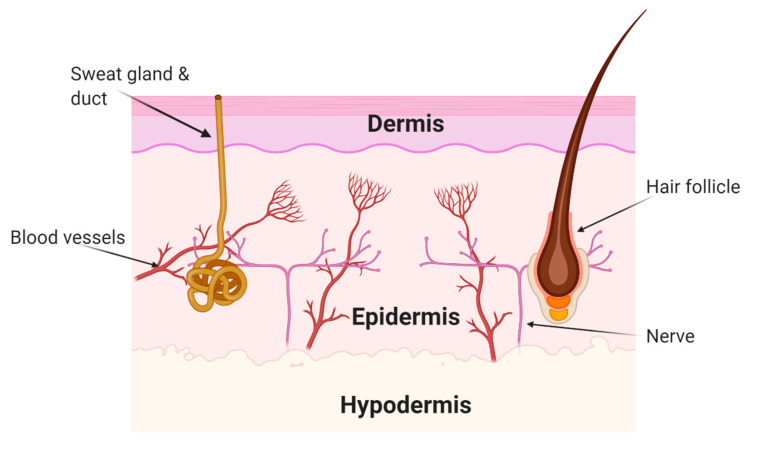
Illustrative representation of the anatomy of human skin.

The skin is the largest organ in the human body, comprising of three distinct layers: the dermis, epidermis, and hypodermis Figure 3. The external layer of skin, the epidermis, has the critical role of protecting the body’s internal environment from the external environment, preventing contamination and infection from pathogenic organisms. The intermediary layer, the dermis, is where the nerves, blood vessels, sweat glands and hair follicles can be found, and finally, the hypodermis is the lower level of the skin, which is where fat is located [91].

The integrity of the skin is paramount to the overall function of the organ, if this integrity is in any way impeded by either intrinsic factors, such as nutritional issues, and illnesses including diabetes or vascular disease; other ailments that cause an impediment to the integrity of the skin; extrinsic factors, such as surgical site wounds, accidents that cause the skin to be cut or pressure sores from immobility. When an individual is subjected to an incident that results in a large proportion of their skin being damaged, this can lead to a cascade effect of localised function impairment, fluid loss/dehydration, necrosis, infection and, in severe cases, limb loss or death [92]. The order in which wound healing occurs follows an exceptionally complex physiological cascade of biological activity that follows many orderly steps that often overlap. The rate at which this occurs is influenced by several factors, such as the size, type, depth of wound and whether there is an infection already present [93]. The result is the restoration of the skin barrier to prevent further infections and fluid loss.

Delays in wound healing can be caused by both endogenous and exogenous microorganisms invading the site and causing infection. This infection can cause significant delays in wound healing by causing chronic non-healing wounds with a high level of inflammation and pain. Infections of wound sites can be attributed to the uncontrolled proliferation and invasion of opportunistic pathogenic microorganisms, such as the *Streptococcus* spp., *S. aureus* and *B. subtillis*, but also, and the focus of this review, fungi, which include *Candida albicans* and *Aspergillus niger* [93,94,95,96]. These pathogens usually make up the natural polymicrobial skin flora but will proliferate when conditions are favourable to their growth. If these micro-organisms can invade a wound site, the chances of delayed wound healing are significantly increased, due to potential nosocomial infections, including candidemia and invasive mycotoxicosis [96].

Although bacterial cellulose provides a physical barrier, which impedes microorganisms from reaching the wound site; in its indigenous form, bacterial cellulose does not possess any anti-microbial properties. In order to increase the efficiency of bacterial cellulose to be used as a treatment option for wounds, specific modifications must be made by either altering the structure of the cellulose matrix, or by the addition of secondary components into the structure to confer an assortment of biological activities; including antimicrobial agents, anti-inflammatory drugs, and growth factors.

The American Burn Association reported in 2018 that there were 490,000 burn-related illnesses which required medical treatment; this included 50,000 long-term hospitalisations for chronic injuries [97]. While the survival rate of severe burns has increased significantly to 97%, they are still complicated and time-consuming to manage effectively. This is due to frequent dressing changes, prolonged hospital stays and, often, very invasive and costly treatments, such as skin grafts. The current method for treating burns and the prevention of infection is the immediate debridement of necrotic tissue, or tissue that is severely damaged which is then followed by a total coverage of the affected wound site with traditional dressings, such as gauze or other expensive hydrogel-based dressings [98]. Additionally, cancer lesions also require continual robust wound management regimes, especially in cases of basal cell carcinoma, or other lesions which have resulted from radiotherapy or chemotherapy, which are often highly vascularized with a severe degree of necrosis, resulting in large amounts of exudate [99].

### 5.2. Wound Dressings

Although bacterial cellulose possesses unique physical and chemical properties, which can be exploited as scaffolds for novel wound dressings, the natural features of the cellulose do not meet all of the expected requirements by the current wound-dressing market. It is now expected that wound dressings have a bio-functional action which aid in the healing process. The most common complication that arises during wound healing involves the contamination of pathogenic organism that cause infections and inflammation, which subsequently results in necrosis and highly delayed healing [100].

Correct moisture control is usually one of the first factors that helps to increase the healing rate of a wound. It also provides a protective barrier against pathogens which would typically cause infections and helps to decrease swelling and pain for the patient. Furthermore, the water-absorbing and holding properties of bacterial cellulose allow for a myriad of liquid pharmaceuticals and other bio-functional compounds to be inserted into the wound dressing [101]. Due to the high water content of bacterial cellulose, the humidity at the wound site is maintained, which prevents the wound dressing from dehydrating and becoming affixed to the actual wound bed, thus preventing further pain and discomfort to the patient during dressing changing, and preventing further trauma to the wound by disturbing the sanguineous crust [102].

As mentioned previously, BC has a high water holding capability, allowing for a moist atmosphere at the site of injury, which is critical in healing, while the absorbent properties allow exudate from the wound to be wicked from the site. Due to the complex structure found in the bacterial cellulose matrix, the large number of hydroxyl groups is capable of high binding volumes of water, with the additional hydrogen bonding of water increasing this limit. This bonding of water is responsible for the flexibility and malleability of bacterial cellulose, while unbonded water can penetrate and diffuse freely out of the cellulose matrix. It is this free water that is responsible for maintaining hydration levels at the wound site, which ensures rapid re-epithelialisation [103].

Bacterial cellulose also contours to the undulating surface of the skin, providing a uniform covering—even in areas that are usually difficult to dress, such as in the groin and neck. This method of producing wound dressings has been so successful that products, such as Biofill^®^, have been developed [103]. Bacterial cellulose also protects healing wounds due to the thickness of the dressing, which minimises the risk of further trauma to the injury. This protection further aids in the promotion of angiogenesis and rapid wound healing [44,103,104].

Although the intrinsic qualities of bacterial cellulose can be exploited as scaffolds for novel wound dressings, the natural features of the cellulose do not meet all of the expected requirements by the current wound-dressing market. It now expected that wound dressings have a bio-functional action, which aids in the healing process. The most common complication that arises during wound healing involves the contamination of a pathogenic organism that causes infections and inflammation, which subsequently results in necrosis and highly delayed healing [94].

Naturally, derived biopolymers present an extremely attractive application in the area of health care, including wound management, in comparison to synthetic polymers. This is because of the low cytotoxicity and biodegradability, along with the potential to be biofunctionalised with antimicrobial agents to prevent direct infection at the wound site [105,106].

Overexposure of the wound bed to the wound exudates causes the damaged tissue to heal more slowly and encourages necrosis. Therefore, all exudates in and around the wound should be removed by means of appropriate drainage methods. This makes up another main criterion for a wound dressing, where the drainage capacity of the dressing should be high enough and appropriate to the size and type of the wound on which it will be placed [107]. For all types of skin lesion, there is a gold standard that wound dressings should strive for. The ideal wound dressing must maintain a steady moisture level at the wound site, which means the dressing must be able to absorb excess exudate, but equally must be able to release moisture when the wound site begins to dry.

**Figure 4:**
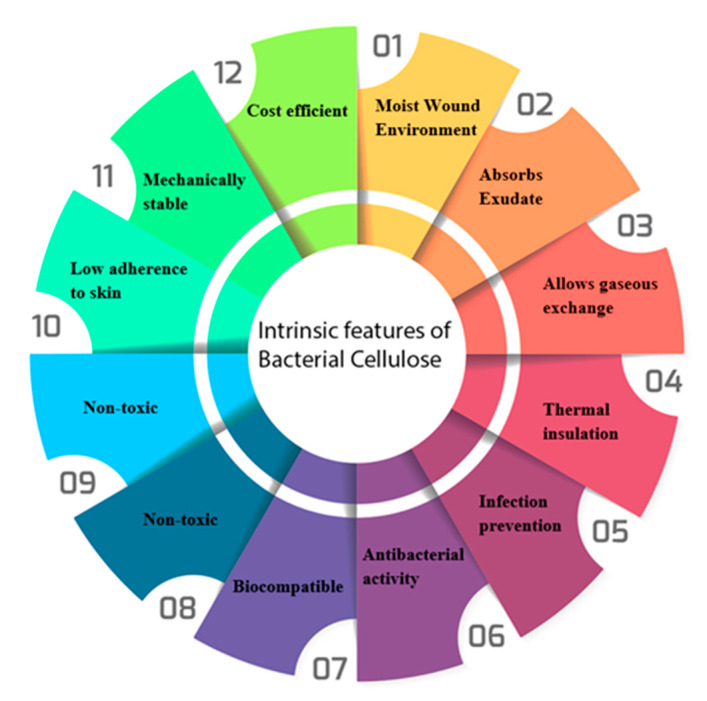
Core intrinsic properties that wound dressings should meet in order to be both commercially and therapeutically successful.

There should also be an adequate gaseous exchange between the wound bed and the atmosphere to allow for the oxygenation of the lesion site. This not only aides in the revascularization of the wound, but also prevents anaerobic pathogens from forming biofilms. Additionally, the wound dressing should provide thermal stability to the wound. This not only maintains a constant temperature, which is ideal for wound healing, but it will also help reduce pain and rubor for the patient. Moreover, the wound dressing should be non-toxic with a high level of mechanical stability with minimal adherence to the skin, which ensures that the dressing does not cause the patient any further trauma or discomfort during the healing process [108]. The properties that wound dressings should possess are summarised in Figure 4. Bacterial cellulose would benefit from further improvement in mechanical properties for use as a wound dressing.

Many different methods have been developed to optimise topical biofunctionalised wound dressings with different constituent compositions. Various compounds, which have been incorporated into bacterial cellulose, vary from small organic molecules to large macromolecules and complex synthetic polymers.

The use of bacterial cellulose has already been successfully used in wound care applications, which has highlighted its potential to become a high-value material in the field of health care and biotechnology, which also coincides with the increased interest in tissue and organ regeneration research, along with antimicrobial resistance. Bacterial cellulose has demonstrated that, through its unique properties, it can meet all of the expectations that a 21st-century wound care product requires.

### 5.3. Currently Marketed Bacterial Cellulose Wound Dressings

Bacterial cellulose is not routinely disseminated throughout the commercial healthcare sector as a wound dressing, despite the multitude of studies, which have been demonstrated with appropriate pre- and post-biosynthesis modifications, which enhance the overall properties of the BC to meet the specific requirements of wound dressings—for example, increased antimicrobial properties, the re-epithelialisation of wounds and shorter healing times. However, there are a few select manufacturers, located in Poland, Brazil and America, who are exploiting bacterial cellulose as a viable material for wound dressings [109]. Lohmann & Rauscher has produced the only drug-activated bacterial cellulose wound dressing by incorporating polyhexamethylene biguanide (a disinfectant) into the cellulose structure under the trade name Suprasorb^®^ X+PHMB.

Companies, such as Biofill™ and Dermafill™, have also produced bacterial cellulose wound dressings, intending to use them as temporary skin substitutes in the treatment of burns and ulcers with patients, stating that, upon application, the dressings provided the immediate cessation of some pain and discomfort. Not only have wound dressings been developed from bacterial cellulose, but dental grafts/implants with bioactive properties have also been produced—for example, Gengiflex^®^, which reduces inflammation and pain post-dental surgery, and encourages the regrowth of periodontal tissues [98]. Other examples of wound dressings and their applications include products from Vuelo Pharma, such as Membracel^®^, which is intended to be used as temporary skin in the treatment of venous leg ulcers and lacerations; the Xylos Corporation developed xCell^®^, which was also established to treat venous leg ulcers with the added activity of accelerating the granulation and autolytic debridement of necrotic tissue. Innovatec produced Nanoskin^®^, which is capable of replacing blood and lymphatic vessels, treating abrasions and epidermolysis bullosa; Nanoderm Ag, developed by the Axcelon Biopolymers Corporation, which is used as an antimicrobial wound dressing due to the silver ions incorporated into the dressing [98].

As a result of research conducted at Lodz University of Technology, Poland, the company S2Medical AB, Sweden began the wide-scale production of bacterial cellulose wound dressings under the brand name EpiProtect^®^. The main target of this dressing was for burn wounds where complete healing of first- and second-degree burns was complete after 28 days of continual dressing with EpiProtectt^®^, in comparison to 32 days with silver sulphadiazine and gauze [109].

## 6. Bacterial Cellulose Composites in Wound Dressings

Materials, that combine at least two different materials, which have a defined interface between them. The composite can acquire specific properties from the constituent materials. Usually, composites are produced to provide the original material with qualities that it did not previously have by itself, and of which are necessary for a particular function. The addition of a material to the base component is called the reinforcing phase and may exist in many forms. These fibres, particles, sheets or cells are disseminated through the original material. The acquired properties of the composite depend on the two materials which are used, and bonding that occurs between then at the interface.

There are four main methods by which compounds can be loaded in the cellulose matrix, these are during the synthesis of cellulose in the growth vessel; post-synthesis via saturation; chemical modification once the cellulose has been processed and purified; finally, through the genetic manipulation of the cellulose-producing organism. To determine the best option for incorporating secondary materials into the cellulose, the physiochemical properties of the compound, such as solubility, stability, molecular size and working concentration needs to be ascertained. These properties need to be determined so the correct loading procedure can be carried out as per the nature of bacterial cellulose, be it wet, dried, or lyophilised [110].

### 6.1. Polysaccahrides

Chitosan is a highly hydrophilic compound, which, when incorporated into bacterial cellulose, promotes a larger quantity of water to be absorbed in comparison to pure bacterial cellulose [38,40,41]. As the chitosan was a secondary component to the bacterial cellulose, the overall porosity was diminished; however, the WHC was increased as a direct result of the chitosan forming hydrogen bonds simultaneously with water and cellulose microfibrils during cellulose synthesis. Moreover, the WRR was increased due to the decrease in porosity [111]. In addition, when chitosan decomposes, N-acetyl-β-D-glucosamine, which prompts the proliferation of fibroblast formation and collagen accumulation, is produced. However, due to the high cost, along with the brittleness of dry films of chitosan, its applications on its own are limited, which has led to the incorporation of the compound into bacterial cellulose [112].

Following this research, Wichai et al., (2019) were able to synthesise an alginate and chitosan bacterial cellulose composite following treatment with hydrogen peroxide. The composite showed all of the mechanical properties that are desired in a wound dressing, and also adequate rehydration properties, which allowed the dressing to be used in absorbing excess exudate. It was later confirmed that the alginate–chitosan–bacterial cellulose wound dressing was also able to release drugs in a controlled manner [112].

### 6.2. Natural and Synthetic Polymers

Bacterial cellulose, which incorporates polymers, is revolutionising the field of biomaterials by incorporating several types of plasticisers within the cellulose matrix. Glycerin was incorporated into the bacterial cellulose to act as a plasticiser and was observed to have a moisturising effect on the skin of burn patients. It was also shown to have biological compatibility with a high level of malleability. Due to these factors, it was concluded that this composite would be ideal in treating dry skin conditions, such as psoriasis, as well as burn wounds [113].

The rate and quantity of water which bacterial cellulose can absorb and retain can also be influenced considerably by integrating anion-exchanged membranes, such as poly-2-aminoethyl-methacrylate (PAEM). The bacterial cellulose-PAEM composite shows that the swelling capacity of the cellulose could be increased by 6200% in comparison to pure bacterial cellulose, which has a swelling ratio of 100% [114]. Additionally, the swelling behaviour observed in this study was concluded to be a result of the conjugation of the hydrophilic PAEM with the bacterial cellulose, which prevents the structure of the cellulose matrix from being destroyed under high water loading conditions as a result of the cellulose fibres breaking.

Moreover, other bacterial cellulose and acrylic hydrogel composites showed an increase in swelling ratios of up to 4000%. The study also confirmed that the bacterial cellulose-acrylic wound dressings promoted enhanced re-epithelialisation and the proliferation of fibroblasts in burn patients in comparison to pure bacterial cellulose as a result of the occurrence of ammonium functional groups in the structure of PAEM, this was also reinforced by additional nanocomposites converting the ammonium groups into potential biocidal agents [44].

Qiao et al., (2018) produced a bacterial cellulose-polyvinyl acrylamide composite that displayed a uniformly porous matrix with improved mechanical properties. This was due to the cross-linking of cellulose with PVA molecules, forming a robust hydrogel [72]. Nguyen (2019) and Aydogdu (2019) showed that 8% polylactic acid could be utilised as a coating agent on bacterial cellulose, which displayed high moisture uptake, high burst and tear ratios and prolonged swelling indices [115,116].

The polylactic acid coating also improved the overall mechanical qualities of the cellulose, maintained the moisture levels within the hydrogel and improved the surface porosity [116]. In addition to this, Sun et al., (2018) investigated methods to improve the characteristics of dry bacterial cellulose. This was done by comparing the additions of glycerol and polyethene glycol to the synthesis of cellulose. It was demonstrated that, not only did the glycerol and polyethene glycol completely encase each microfiber of cellulose, but upon drying, the two respective plasticizers expanded the cellulose matrix creating more free interstitial space [117]. Moreover, the durability of the materials increased drastically in comparison to pure bacterial cellulose. It was also noted that, upon re-wetting, the two composite materials had higher water retention and water absorption ratios, compared to the untreated cellulose. Furthermore, due to the expanded interstitial spaces in the modified composites, the water vapour transmission rates were shown to be 2900 g/m^2^/day [117].

### 6.3. Nanoparticles

In addition to hydrophilic polymers, metal nanoparticles can also be amalgamated into the bacterial cellulose matrix. Magnetite nanoparticles can be dispersed throughout the extracellular matrix during biosynthesis in the growth media [68]. This study showed that the presence of nanoparticles in the growth media had little effect on the production of cellulose. Through nanoparticles into the growth media during the biosynthesis of cellulose, it was possible to improve the overall physicochemical properties of the material [54,80,95,114,117,118,119]. Silver nanoparticles exhibit antimicrobial properties with minimal resistance, as seen in antibiotic resistance, and when they were integrated into bacterial cellulose (BS:Ag), following the Kirby–Bauer disk diffusion assay, the BC:Ag was shown to have an average zone of inhibition of 13 mm against *S. aureus* and *P. aeruginosa* [54].

There are several forms of silver nanoparticles which can be inserted into the bacterial cellulose; these can take the form of zeolites, silver sulfadiazine or silver nitrate. These compounds have shown antimicrobial action for many of the most potent pathogenic organisms. The bacterial cellulose-silver nanoparticle composites are proven through MTT cytotoxic assays against U251, MSTO and Panc 1 cell lines to be non-toxic. Additionally, due to the controlled release of silver ions, damage to mononuclear blood cells was minimal [54,119,120,121,122].

Composites that contain silver nanoparticles remain transparent, which allows for the continual visualization of the wound site without the need to remove the wound dressing [120]. Mohseni et al., (2016) conducted comparative studies on rats, which demonstrated that, when wounds were treated with silver nanoparticle-bacterial cellulose, loaded with amikacin and ceftriaxone, presented with more significant wound healing with the inhibition of pathogenic organisms, without hindrance to re-epithelization [116]. In addition to silver nanoparticles, gold nanoparticle-bacterial cellulose composites have been reported to have a higher biocidal effect in comparison to most antibiotics used against Gram-negative bacteria. This was reported alongside the fact that the composite retained physicochemical qualities, such as water uptake, tensile strength and low adhesion [114].

Mathew et al., (2018) studied the effects of immobilising smaller nanoparticles of silver in montmorillonite clay, which again, when combined with bacterial cellulose, showed high levels of antibacterial action [117]. The addition of the montmorillonite clay also provided the bacterial cellulose with higher water retaining and mechanical properties, and caused the silver ions to be released more slowly. Regardless of the slow-release ratio, the small particle size of the silver nanoparticles allowed for more the effective penetration of bacterial cells [121]. This can be attributed to the high electrostatic affinity the nanoparticles have for the bacterial cells, which allowed for a higher convergence of nanoparticles on the bacterial membrane, which had the overall effect of prevent the cell from growing.

Aboelnaga et al., (2018) successfully conjugated silver nanoparticles into bacterial cellulose by first treating the bacterial cellulose with dimethyl sulfoxide that was successively immersed in a solution of diaminobutane and then in a solution of dimethyl sulfoxide, silver nitrate and sodium acetate [120]. Amine groups, which were created on the bacterial cellulose surface, acted as anchoring points for the silver nanoparticles to bind. The resulting bacterial cellulose-silver nanoparticle composite displayed improved retention time of the nanoparticle while maintaining a very high level of antibacterial activity against *E. coli* [121,122]. Furthermore, 2,2,6,6-Tetramethylpiperidinyloxy was utilised as an intermediate during the oxidation process, which used TEMPO, sodium hypochlorite and sodium bromide to obtain six-carbon carboxylate, functional groups. The modified bacterial cellulose was then functionalised with silver nanoparticle through an ionic exchange in silver nitrate solution. The subsequent bacterial cellulose-silver nitrate hydrogels showed very low cytotoxicity to fibroblasts with cell viability of 95.2% after 48 h exposure with 100% biocidal activity against *S. aureus* and *E. coli* [123].

### 6.4. Metal Oxides

Additionally, Khan (2015) indicated that the titanium dioxide-bacterial cellulose composite also had high cellular adhesion and caused the upregulated proliferation of fibroblast without any toxicity to the surrounding cells where the A450 at day 1 was 0.1 and, by day 5, was 0.9 [124].

Chu et al., (2018) used photodynamic therapy to combine C60 isotopes into bacterial cellulose to treat skin cancer. Due to the photodynamic therapy technique, the C60 was homogenously dispersed throughout the fibrillar matrix of cellulose. This study showed that the bacterial cellulose-C60 composite was highly capable of producing large quantities of reactive oxygen species when exposed to light. These oxygen species are highly destructive to bacteria and, as such, inhibit the growth of any pathogenic organisms. The stability of these composites was investigated, which showed minimal activity when kept away from light; however, when exposed to light, the composite showed a reduction in epidermoid carcinoma cells A-143 by 80% [95].

### 6.5. Antimicrobials

There are also strong indications that bacterial cellulose, which has been loaded with antimicrobial agents, such as povidone-iodine, polyhexamethylene biguanide and octenidine showed a 1.5 fold increase in antimicrobial action against both Gram-positive and Gram-negative bacteria, compared to the cotton gauze analogue [48]. Due to the high molar mass, when povidone-iodine was combined with the cellulose, the structure became more compressed with the increased overall compressive strength of the material, while the combination of polyhexamethylene biguanide and cellulose again showed no alteration to the tensile strength of the resulting composite. All three composites showed up to 65% antimicrobial activity against *S. aureus* [48].

Cationic surfactants with antimicrobial activity, such as benzalkonium chloride, are highly active against Gram-positive bacteria and are routinely used in current wound dressings. They were tested by soaking bacterial cellulose for 24 h. It was shown that the loading capacity of the cellulose increased when the concentration of benzalkonium chloride was increased, while 90% of the compound was released within the first 24 h. Following cytotoxicity testing on mononuclear cells, it was shown that over 90% of cells survived, which would allow this material to be used a regenerative material [125].

Low molecular weight biocidal compounds, such as quaternary ammonium compounds, have a positive charge with a highly hydrophobic site. They are highly biocidal due to their membrane penetrating properties and have low toxicity to human cells. More recently, quaternary ammonium compounds were bonded to an 18-carbon fatty acid-di-linoleic acid, tyrosine and ethylenediamine. The subsequent compound, EDA-DLA-Tyr, was loaded onto bacterial cellulose by submerging for 24 h. The resulting composite showed antibacterial activity against *Staphylococcus aureus* and *Staphylococcus epidermidis,* both of which are highly opportunistic microbes, which are responsible for the bulk of nosocomial infections [126].

The addition of amoxicillin into bacterial cellulose was conducted by employing a cross-linking strategy whereby the cellulose was treated with 3-aminopropyl-trietoxysilane to bond aminoalkysilane groups through Si-O-C bonding. The reactive carboxylic group located on amoxicillin was adapted by treating the drug with a carbodiimide crosslinker—EDC/NHS (1-ethyl-3-(3-dimethyl aminopropyl) carbodiimide hydrochloride). Once the amoxicillin had been modified with activated ester groups, it was able to form covalent bonds with the NH_2_ groups in the bacterial cellulose. The resulting bacterial cellulose composite demonstrated up to a 95% reduction in the viability of *C. albicans, E. coli* and *S. aureus* [127].

Wijaya et al., (2017) showed that bacterial cellulose loaded with tetracycline was capable of releasing 82% of all loaded drugs within 48 h of application and demonstrated a 99.9% reduction in the viability of *S. aureus* and *B. subtilis* [128]. The microporous structure and large surface area allow for a large number of active compounds to be retained. These features also directly influence the slow release of these compounds into the target wound site, which allows for a longer-lasting effect. Regardless of the strides in biotechnology, the most frequently used method to load materials into bacterial cellulose follows the immersion and saturation technique. The most commonly reported technique involves freeze-drying purified bacterial cellulose and soaking it in a solution of the active compound.

### 6.6. Anaesthesia and Analgesics

Topical anaesthetics, such as lidocaine, along with non-steroidal anti-inflammatory drugs such as ibuprofen and diclofenac, can also be combined with bacterial cellulose as the opposing hydrophilicity, provided a different basis for a wound dressing, which relies on water content. Bacterial cellulose was soaked in 5% lidocaine solution and subsequently soaked in an ethanol solution [129]. Following diffusion assays, lidocaine was released at a low steady rate, while ibuprofen release at three times the rate of commercially available dressings with similar concentrations of [88]. Diclofenac was loaded onto bacterial cellulose by soaking the cellulose in glycerol containing 5% diclofenac, which displayed a swelling ratio six times higher than in pure bacterial cellulose. This indicates that bacterial cellulose loaded with non-steroidal anti-inflammatory drugs are more advantageous in comparison to their counterpart transdermal dressings, as it was shown that there was a 9% increase in diclofenac release from loaded BC. [56,57,130].

### 6.7. Proteins, Enzymes and Amino Acids

With regard to enzyme incorporation, the enzyme must first be immobilised. Bacterial cellulose is an ideal candidate for enzyme loading, as a simple submersion technique will ensure the proteins bind to the material without causing any inhibition to the activity of the enzyme. The immersion incorporation of serum albumin was conducted by soaking lyophilised bacterial cellulose in the solution. The proteins remained fully functional due to the freeze-drying process altering the structure of the bacterial cellulose, which also aided in the release of the proteins [131,132]. Lipase proteins were also investigated through the repeated absorption technique, where repeated immersion and drying took place to obtain maximum loading of the protein. The bacterial cellulose-lipase complex was activated using a glutaraldehyde-reticulating agent, which resulted in a 90% efficiency of the immobilised enzyme. After testing, it was shown that 62% of the lipase retained its activity after 15 repeated uses on animal wounds, regarding which it is highly suggestive that the two-step technique is cost-effective and appropriate for industrial production [78].

The conjugation process did, however, impede the flexibility of the cellulose but did improve the overall access to the enzyme, which resulted in a high antimicrobial profile. The composite was successful in reducing the growth of Gram-positive bacteria with cytotoxic indices, which were deemed acceptable for applications in wound dressings [79].

One developing area of the bioactivation of bacterial cellulose arises from the conjugation of two active compounds within the cellulose matrix. This was investigated by the incorporation of silk sericin, which enhances collagen I, which is essential in the re-epithelialisation of wounds, and the antiseptic compound polyhexamethylene biguanide (PHMB). Following conjugation via immersion, it was noted that silk sericin must be loaded before polyhexamethylene biguanide for PHMB to retain antimicrobial activity [126].

E-poly-L-lysine, which is a broad-spectrum anti-microbial, which can be found in the first step of the innate immune system in various organisms, is non-toxic, biodegradable, water-soluble and disrupts the cellular membrane of bacteria, which makes it an ideal candidate to be used in a wound dressing. E-poly-L-lysine, with a low molecular weight of approximately 5k Da, was inserted into bacterial cellulose by firstly exposing the homopolymer to carbodiimide and then covalently bonding the E-poly-L-lysine to bacterial cellulose, which has been carboxymethylated. This method resulted in high levels of cytocompatibility to fibroblasts, and also antimicrobial properties towards staphylococcus for up to 30 days [133].

A glycosaminoglycan called hyaluronan, which is present in the synovial fluid between joints has been shown to have the capacity to bind large quantities of water, which aids in tissue hydration. The rheological properties of hyaluronan, which increase fluid viscosity, provide the tissue with mechanical resistance to damage. Hyaluronan is well documented for its anti-apoptotic and pro-angiogenic properties, which is highly beneficial in the re-epithelialisation of wounds with a reduction in scar formation [134].

During the skin healing process, it is known that a rapid peak in hyaluronan levels is strongly associated with inflammation, mesenchymal and epithelial cell migration and proliferation, and cytokine induction. Hyaluronan extends from the cell surface to form networks called cables, which act as nets which entrap platelets and leucocytes, resulting in modulation of inflammation. High molecular weight hyaluronan was combined with bacterial cellulose, which was shown to stimulate the healing process. The composites were produced by means of the submersion technique and enhanced the overall thermal stability of the bacterial cellulose, while lowering the available surface area, average pore volume and tensile strength [134,135].

Table 3 shows a summary of the additives which can be incorporated into BC along with the resulting property it induces.

## 7. Limitations and Future Advancements

Market Research Reports (2019) has stated that, by 2023, the microbial cellulose market will have increased by 14.8% with a market value of $570 million in America alone, indicating that the interest in utilising bacterial cellulose for a variety of applications, including as a wound management system, has become far more attractive to large pharmaceutical/biotechnology companies [136].

Bacterial cellulose is already being used to advance bio-material utilisation and has seen increased applications as 3D printing compounds. One advantage of using bacterial cellulose as a 3D printing material is the unlimited variations of wound dressing design and functionality. This is because the overall structure of the wound dressing can be precisely designed to fit a specific purpose; be it specialised drug loading, that requires an extremely precise pore size, or the development of wound dressings with incorporated biosensors, which would allow the wound dressing to provide clear indications of potential infections or the current progress of healing [137]. This can be achieved by combining light-sensitive pH materials into the matrix that will fluoresce under UV light, according to the pH of the wound, as the cellulose dressing is transparent, which can be done without having to remove the dressing and thus allows the healing process to be unhindered [138,139,140].

Bacterial cellulose has been further adapted to measure vital signs of a wound, such as temperature. This was achieved by printing silver ink onto the bacterial cellulose-polyurethane dressings, which were then attached to a computer that was able to read the temperature of the wound site directly. This is particularly important, as an increase in temperature at the wound site is indicative of an infection and can be monitored continuously. The wound dressing itself was impregnated with glycerol and alginate, which aides in the re-epithelialisation of the wound and prevents the formation of sanguineous crusts by keeping the newly formed tissue hydrated and malleable [141].

Personalised medicine is approaching commonplace practice in medical settings at astonishing speed, especially with a deeper understanding that individual circumstances require individual treatment regimens with specific factors. An example of this centres around diabetes, with each patient reacting in individualistic ways. Any formation of ulcers and wounds, as a result of this illness, have to be treated following the patients’ parameters. A thorough assessment of the patient’s comorbidities, health and nutrition are all highly important factors in the formulations of a treatment regimen [142]. When these parameters are taken into account, the ability to design and produce individual bacterial cellulose wound dressings that are capable of releasing dose-specific medications directly to the wound site, all whilst monitoring the healing process of the wound in real-time, without the need to change the dressing, appears to be a highly challenging task, but well within the scope of current biotechnologies.

This review has highlighted that bacterial cellulose has a wide-reaching gamut of applications in biotechnological and biomedical applications. Within these applications, it is apparent that the cellulose matrix is integral to its functionality as a novel biomaterial, allowing for additional components to be loaded, which ultimately defines its intended use, from stem cell-loaded wound dressings to conductive nanoparticles for use as electrical switches. The examples given in this review indicate that research, which is currently being conducted in bacterial cellulose as a biomaterial, frames it as an ideal aspirant to be used instead of conventional synthetic materials or analytical techniques [143].

However, there is some doubt that has been raised, as not all results obtained are cohesively aligned with the predictions of researchers. Numerous issues which have arose, especially around biomedical applications, which revolve around the storage, handling and production of bacterial cellulose derivatives. The technique of “bioprinting” BC into useful bioactivated membranes has elucidated an effect where the finished printed item has a considerably lower tensile strength than what was estimated [144]. This was further compounded by difficulties in sterilising the BC membranes by autoclaving for use as tissue grafts and wound dressings. Although the BC itself is highly stable under high heat, the bioactive components of the material are usually temperature sensitive and become inactive, rendering the material ineffective for its targeted use. This can be overcome by alternative methods of sterilisation, such as radiation, but these are accompanied by their own inherent problems, such as the denaturation of enzymes and proteins [144].

For BC materials to be considered a viable option within the biomedical field, it is crucial that a satisfactory sterilisation method is developed as unsterile surgical implants or wound dressings could introduce significant levels of contamination. Further issues for bacterial cellulose as a drug delivery material revolve around the difficulty in modulating drug release, and irregular loading patterns of pharmaceuticals. Improved protocols and the development of novel processing techniques should be explored in order to overcome these issues which currently impede the widespread use of bacterial cellulose as a biomedical material [145].

It is clear that there are many advantages and disadvantages to the utilisation of bacterial cellulose as biomaterial, especially for biomedical applications; the latter urgently needs to be addressed before BC-based biomaterials can be routinely employed [146]. Nevertheless, it is clear from global research that there is a high level of interest in developing biomaterials from bacterial sources, compared to vegetal sources, because of the cost-effective production of pure cellulose fibres, which can be further modified.

## 8. Concluding Remarks

In conclusion, bacterial cellulose has been identified as a highly adaptable material to produce medically relevant materials, such as wound dressings, composites, dental grafts, and gels, which all have distinctive characteristics that are suited to their role. These materials provide a viable and cost-effective alternative to combat the use of petroleum-based analogues. To this end, bacterial cellulose is highly biodegradable, with outstanding physicochemical properties, because of the nanofibrillar matrix. The fact that this material also exhibits no toxicity in nearly all applications, due to the high level of native purity, would allow for this dressing to be directly utilised. Bacterial cellulose biomaterials have been investigated in a multitude of applications focused on biomedical purposes and the development of wound dressings for moderate to severe wounds. These investigations have led to the elucidation of the enormous potential of these composites to be effective in treating wounds, despite the lack of in vivo studies. The points raised in this review address the efforts and advancements to optimise the use of bacterial cellulose in the production of bioactivated wound dressings. Nonetheless, none of these strategies currently meet all of the issues that need to be addressed in order to make this material a genuinely viable option for a wound dressing; primarily, the high cost of scaling up production.

## Figures and Tables

**Table 2:** Summary of various applications in which bacterial cellulose has been used.

Application	Description	Reference
Wound dressing	Low cytotoxicity, swelling ratio, physicochemical and mechanical properties of BC make it an ideal biomaterial for wound dressings	[38,39,43,44,45,46,48,50,51,53,54]
Antimicrobial activity	Capable of being biofunctionalized with various antimicrobial agents	[33,37]
Tissue regeneration	Intracellular matrix of BC allows it to be used a scaffold for tissue growth	[4,5,31,32,60,63,66,67,68,69,70]
Drug delivery	Water holding capabilities allow for pharmaceuticals to be loaded	[10,48,56,57]
Vascular grafts	Elasticity and tensile strength allow it to be used as artificial blood vessels	[63,72]
Surgical reconstruction	Can be used as a biodegradable alternative to synthetic surgical consumables	[60,61,62,63,64,65,66]
Emulsion stabiliser	Fractionated BC can be used as an emulsifier in oil-water mixtures	[86]
Dietary supplement	BC is pure fiber and can be eaten to aide digestion	[85,87]
Enzyme immobilisation	Surface modifications allow for enzymes to be immobilised	[72]
Filtration system	Nanofibrillar matrix and carbon coatings allow BC to be used an effective filter	[79]
Conductive material	Modification with metal nanoparticles make the BC conductive for use in nanowires	[76,78,81,82,83,84,85,86]
Biosensors	Biofunctionalised BC can be made into patches that are sensitive to biochemical tests	[76,77,78]

**Table 3:** Comparative table of bacterial cellulose composites showing the property achieved when various active compounds are amalgamated.

Active Compound in Bacterial Cellulose Composite	Property	Reference
Polyhexanide	Antimicrobial activity	[126]
Octenidine dihydrochloride	Antimicrobial activity	[48,52]
Benzalkonium chloride	Antimicrobial activity	[126]
Tetracycline	Antimicrobial activity	[47,55,62]
Amoxicillin	Antimicrobial activity	[127]
Povidone iodine	Antimicrobial activity	[48]
Antimicrobial peptides	Antimicrobial activity	[133]
Lysozyme	Antimicrobial activity	[37]
Dehydrogenative polymers	Antimicrobial activity	[5]
Silver Nanoparticles	Antimicrobial activity	[54,115,116,117,118,119,120,121,122]
Chitosan	Antimicrobial activity	[38,40,41,51,107,108,129,131]
Zinc Oxide	Antimicrobial activity	[80]
Antibiotics	Antimicrobial activity	[101,120]
Gold nanoparticles	Antimicrobial activity	[114]
Diclofenac/Ibuprofen	Anti-inflammatory	[56,57]
Lidocaine/Benzocaine	Analgesia	[129]
Silk sericin	Angiogenesis and re-epithelisation	[126]
Mesenchymal stem cells	Angiogenesis and re-epithelisation	[4]
Epidermal keratinocytes	Angiogenesis and re-epithelisation	[44]
Dermal fibroblasts	Angiogenesis and re-epithelisation	[44,67,71]
Polyvinyl alcohol	Mechanical performance	[41]
Plasticisers	Mechanical performance	[109]
Sodium alginate	Mechanical performance	[98,108]
Polylactic acids	Physicochemical properties	[112]
Hyaluronic acid	Physicochemical properties	[134,135]
Hydrolysed gelatine	Physicochemical properties	[43,98,107]
Acrylic acid	Faster wound healing	[44]
Magnetite	Faster wound healing	[68,81]
Radioactive isotopes	Cancer wound therapy	[95]

## Data Availability

The data presented in this study is openly available.

## References

[1] Park M.-A., Lee S.-G., Choi K.-H., Cho B.-U. (2019). Effects of Surface Coating of Cellulose II-based MFC on Paper Properties. J. Korea Tech. Assoc. Pulp Pap. Ind..

[2] Fernandes S.N., Almeida P.L., Monge N., Aguirre L.E., Reis D., De Oliveira C.L.P., Neto A.M.F., Pieranski P., Godinho M.H. (2017). Cellulose Nanocrystals: Mind the Microgap in Iridescent Cellulose Nanocrystal Films. Adv. Mater..

[3] Abba M., Abdullahi M., Nor M.H.M., Chong C.S., Ibrahim Z. (2018). Isolation and characterisation of locally isolated Gluconacetobacter xylinus BCZM sp. with nanocellulose producing potentials. IET Nanobiotechnol..

[4] Shpichka A.I., Butnaru D., Bezrukov E.A., Sukhanov R.B., Atalaa A., Burdukovskii V.F., Zhang Y., Timashev P. (2019). Skin tissue regeneration for burn injury. Stem Cell Res..

[5] Sajjad W., Khan T., Ul-Islam M., Khan R., Hussain Z., Khalid A., Wahid F. (2019). Development of modified mont-morillonite-bacterial cellulose nanocomposites as a novel substitute for burn skin and tissue regeneration. Carbohydr. Polym..

[6] Singhsa P., Narain R., Manuspiya H. (2018). Physical structure variations of bacterial cellulose produced by different Komagataeibacter xylinus strains and carbon sources in static and agitated conditions. Cellulose.

[7] Krasteva P.V., Bernal-Bayard J., Travier L., Martin F.A., Kaminski P.-A., Karimova G., Fronzes R., Ghigo J.-M. (2017). Insights into the structure and assembly of a bacterial cellulose secretion system. Nat. Commun..

[8] Jang W.D., Kim T.Y., Kim H.U., Shim W.Y., Ryu J.Y., Park J.H., Lee S.Y. (2019). Genomic and metabolic analysis ofKomagataeibacter xylinusDSM 2325 producing bacterial cellulose nanofiber. Biotechnol. Bioeng..

[9] He F., Yang H., Zeng L., Hu H., Hu C. (2020). Production and characterization of bacterial cellulose obtained by Gluconacetobacter xylinus utilizing the by-products from Baijiu production. Bioprocess Biosyst. Eng..

[10] Römling U., Galperin M.Y. (2015). Bacterial cellulose biosynthesis: Diversity of operons, subunits, products, and functions. Trends Microbiol..

[11] Badshah M., Ullah H., Khan A.R., Khan S., Park J.K., Khan T. (2018). Surface modification and evaluation of bacterial cellulose for drug delivery. Int. J. Biol. Macromol..

[12] Thiruvengadam V., Vitta S. (2017). Bacterial cellulose based flexible multifunctional nanocomposite sheets. Cellulose.

[13] Prosvirnikov D.B., Safin R.G., Zakirov S. (2018). Microcrystalline Cellulose Based on Cellulose Containing Raw Material Modified by Steam Explosion Treatment. Solid State Phenom..

[14] Volova T., Prudnikova S.V., Sukovatyi A.G., Shishatskaya E.I. (2018). Production and properties of bacterial cellulose by the strain Komagataeibacter xylinus B-12068. Appl. Microbiol. Biotechnol..

[15] Gorgieva S. (2020). Bacterial Cellulose as a Versatile Platform for Research and Development of Biomedical Materi-als. Processes.

[16] Negut I., Grumezescu V., Grumezescu A.M. (2018). Treatment Strategies for Infected Wounds. Molecules.

[17] Rahman M., Netravali A. (2016). Aligned Bacterial Cellulose Arrays As “Green” Nanofibers for Composite Materials. ACS Macro Lett..

[18] Czaja W., Romanovicz D., Brown R.M. (2004). Structural investigations of microbial cellulose produced in stationary and agitated culture. Cellulose.

[19] Thygesen A., Oddershede J., Lilholt H., Thomsen A., Ståhl K. (2005). On the determination of crystallinity and Cel-lulose content in plant fibres. Cellulose.

[20] Ruan C., Zhu Y., Zhou X., Abidi N., Hu Y., Catchmark J. (2016). Effect of cellulose crystallinity on Bacterial Cellu-lose assembly. Cellulose.

[21] Fernandes M., Gama M., Dourado F., Souto A. (2019). Development of novel bacterial cellulose composites for the tex-tile and shoe industry. Microb. Biotechnol..

[22] Du R., Wang Y., Zhao F., Qiao X., Song Q., Li S., Kim R.-C., Pan L., Han Y., Xiao H. (2020). Production, Optimization and Partial Characterization of Bacterial Cellulose from Gluconacetobacter xylinus TJU-D2. Waste Biomass Valorization.

[23] Huang Y., Zhu C., Yang J., Nie Y., Chen C., Sun D. (2014). Recent advances in bacterial cellulose. Cellulose.

[24] Revin V., Pestov N., Shchankin M., Mishkin V., Platonov V., Uglanov D. (2019). A Study of The Physical and Me-chanical Properties of Aerogels Obtained from Bacterial Cellulose. Biomacromolecules.

[25] Ul-Islam M., Khan T., Park J.K. (2012). Water holding and release properties of bacterial cellulose obtained by in situ and ex situ modification. Carbohydr. Polym..

[26] Bandyopadhyay S., Saha N., Sáha P. (2018). Characterization of Bacterial Cellulose Produced using Media Containing Waste Apple Juice. Appl. Biochem. Microbiol..

[27] Liu D., Cao Y., Qu R., Gao G., Chen S., Zhang Y., Wu M., Ma T., Li G. (2019). Production of bacterial cellulose hy-drogels with tailored crystallinity from Enterobacter sp. FY-07 by the controlled expression of colanic acid synthetic genes. Carbohydr. Polym..

[28] Rühs P., Storz F., López Gómez Y., Haug M., Fischer P. (2018). 3D bacterial cellulose biofilms formed by foam templat-ing. npj Biofilms Microbiomes.

[29] Zhai X., Lin D., Li W., Yang X. (2020). Improved characterization of nanofibers from bacterial cellulose and its potential application in fresh-cut apples. Int. J. Biol. Macromol..

[30] Sulaeva I., Henniges U., Rosenau T., Potthast A. (2015). Bacterial cellulose as a material for wound treatment: Proper-ties and modifications. A review. Biotechnol. Adv..

[31] Khan M.U.A., Haider S., Haider A., Razak S.I.A., Kadir M.R.A., A Shah S., Javed A., Shakir I., Al-Zahrani A.A. (2021). Development of porous, antibacterial and biocompatible GO/n-HAp/bacterial cellulose/β-glucan biocomposite scaffold for bone tissue engineering. Arab. J. Chem..

[32] Roman M., Haring A.P., Bertucio T.J. (2019). The growing merits and dwindling limitations of bacterial cellulose-based tissue engineering scaffolds. Curr. Opin. Chem. Eng..

[33] Alavi M. (2019). Modifications of microcrystalline cellulose (MCC), nanofibrillated cellulose (NFC), and nanocrystalline cellulose (NCC) for antimicrobial and wound healing applications. e-Polymers.

[34] Cavalcanti L.M., Pinto F.C.M., De Oliveira G.M., Lima S.V.C., Aguiar J.L.D.A., Lins E.M. (2017). Efficacy of bacterial cellulose membrane for the treatment of lower limbs chronic varicose ulcers: A randomized and controlled trial. Rev. Do Colégio Bras. De Cir..

[35] Grela E., Kozłowska J., Grabowiecka A. (2018). Current methodology of MTT assay in bacteria—A review. Acta Histochem..

[36] Silva W.N., Leonel C., Prazeres P.H.D.M., Sena I.F.G., Guerra D.A.P., Heller D., Diniz I.M.A., Fortuna V., Mintz A., Birbrair A. (2018). Role of Schwann cells in cutaneous wound healing. Wound Repair Regen..

[37] Bayazidi P., Almasi H., Asl A.K. (2018). Immobilization of lysozyme on bacterial cellulose nanofibers: Characteristics, antimicrobial activity and morphological properties. Int. J. Biol. Macromol..

[38] Cacicedo M.L., Pacheco G., Islan G.A., Alvarez V.A., Barud H.S., Castro G.R. (2020). Chitosan-bacterial cellulose patch of ciprofloxacin for wound dressing: Preparation and characterization studies. Int. J. Biol. Macromol..

[39] Volova T., Shumilova A., Nikolaeva E., Kirichenko A., Shishatskaya E. (2019). Biotechnological wound dressings based on bacterial cellulose and degradable copolymer P(3HB/4HB). Int. J. Biol. Macromol..

[40] Piasecka-Zelga J., Zelga P., Szulc J., Wietecha J., Ciechańska D. (2018). An in vivo biocompatibility study of surgical meshes made from bacterial cellulose modified with chitosan. Int. J. Biol. Macromol..

[41] Cazón P., Velazquez G., Vázquez M. (2019). Characterization of bacterial cellulose films combined with chitosan and polyvinyl alcohol: Evaluation of mechanical and barrier properties. Carbohydr. Polym..

[42] Van Zyl E., Coburn J. (2019). Hierarchical Structure of Bacterial-Derived Cellulose and Its Impact on Biomedical Ap-plications. Curr. Opin. Chem. Eng..

[43] Ye S., Jiang L., Su C., Zhu Z., Wen Y., Shao W. (2019). Development of gelatin/bacterial cellulose composite sponges as potential natural wound dressings. Int. J. Biol. Macromol..

[44] Mohamad N., Loh E.Y.X., Fauzi M.B., Ng M.H., Amin M.C.I.M. (2018). In vivo evaluation of bacterial cellulose/acrylic acid wound dressing hydrogel containing keratinocytes and fibroblasts for burn wounds. Drug Deliv. Transl. Res..

[45] Keyes B.E., Liu S., Asare A., Naik S., Levorse J., Polak L., Lu C.P., Nikolova M., Pasolli H.A., Fuchs E. (2016). Impaired Epidermal to Dendritic T Cell Signaling Slows Wound Repair in Aged Skin. Cell.

[46] Swingler S., Gupta A., Heaselgrave W., Kowalczuk M., Radecka I. (2019). An investigation into the anti-microbial properties of bacterial cellulose wound dressings loaded with curcumin:hydroxypropyl-β-cyclodextrin supramolecular inclusion complex An investigation into the anti-microbial properties of bacterial cellulose wound dressings loaded with curcumin:hydroxypropyl-β-cyclodextrin supramolecular inclusion complex. Access Microbiol..

[47] Shao W., Wang S., Liu X., Liu H., Wu J., Zhang R., Min H., Huang M. (2015). Tetracycline hydrochloride loaded regenerated cellulose composite membranes with controlled release and efficient antibacterial performance. RSC Adv..

[48] De Mattos I.B., Nischwitz S.P., Tuca A.-C., Groeber-Becker F., Funk M., Birngruber T., Mautner S.I., Kamolz L.-P., Holzer J.C. (2020). Delivery of antiseptic solutions by a bacterial cellulose wound dressing: Uptake, release and antibacterial efficacy of octenidine and povidone-iodine. Burn.

[49] Junka A., Bartoszewicz M., Dziadas M., Szymczyk P., Dydak K., Żywicka A., Owczarek A., Bil-Lula I., Czajkowska J., Fijałkowski K. (2019). Application of Bacterial Cellulose Experimental Dressings Saturated with Gentamycin for Man-agement of Bone Biofilm In Vitro And Ex Vivo. J. Biomed. Mater. Res. Part B Appl. Biomater..

[50] El-Wakil N., Hassan E., Hassan M., Abd El-Salam S. (2019). Bacterial Cellulose/Phytochemical’s Extracts Biocomposites For Potential Active Wound Dressings. Environ. Sci. Pollut. Res..

[51] Ao H., Jiang W., Nie Y., Zhou C., Zong J., Liu M., Liu X., Wan Y.-Z. (2020). Engineering quaternized chitosan in the 3D bacterial cellulose structure for antibacterial wound dressings. Polym. Test..

[52] Alkhatib Y., Dewaldt M., Moritz S., Nitzsche R., Kralisch D., Fischer D. (2017). Controlled Extended Octenidine Re-lease From A Bacterial Nanocellulose/Poloxamer Hybrid System. Eur. J. Pharm. Biopharm..

[53] Gupta A., Keddie D., Kannappan V., Gibson H., Khalil I., Kowalczuk M., Martin C., Shuai X., Radecka I. (2019). Production and characterisation of bacterial cellulose hydrogels loaded with curcumin encapsulated in cyclodextrins as wound dressings. Eur. Polym. J..

[54] Gupta A., Briffa S.M., Swingler S., Gibson H., Kannappan V., Adamus G., Kowalczuk M., Martin C., Radecka I. (2020). Synthesis of Silver Nanoparticles Using Curcumin-Cyclodextrins Loaded into Bacterial Cellulose-Based Hydrogels for Wound Dressing Applications. Biomacromolecules.

[55] Shao W., Liu H., Wang S., Wu J., Huang M., Min H., Liu X. (2016). Controlled release and antibacterial activity of tetracycline hydrochloride-loaded bacterial cellulose composite membranes. Carbohydr. Polym..

[56] Maver U., Xhanari K., Žižek M., Gradišnik L., Repnik K., Potočnik U., Finšgar M. (2020). Carboxymethyl cellu-lose/diclofenac bioactive coatings on AISI 316LVM for controlled drug delivery, and improved osteogenic poten-tial. Carbohydr. Polym..

[57] Silva N.H., Rodrigues A.F., Almeida I.F., Costa P., Rosado C.F., Neto C.P., Silvestre A.J.D., Freire C.S.R. (2014). Bacterial cellulose membranes as transdermal delivery systems for diclofenac: In vitro dissolution and permeation studies. Carbohydr. Polym..

[58] Hoshi T., Yamazaki K., Sato Y., Shida T., Aoyagi T. (2018). Production of hollow-type spherical bacterial cellulose as a controlled release device by newly designed floating cultivation. Heliyon.

[59] Zmejkoski D., Spasojević D., Orlovska I., Kozyrovska N., Soković M.D., Glamočlija J., Dmitrović S., Matovic B., Tasić N., Maksimović V. (2018). Bacterial cellulose-lignin composite hydrogel as a promising agent in chronic wound healing. Int. J. Biol. Macromol..

[60] Bäckdahl H., Esguerra M., Delbro D., Risberg B., Gatenholm P. (2008). Engineering Microporosity in Bacterial Cellu-lose Scaffolds. J. Tissue Eng. Regen. Med..

[61] Leitão A.F., Faria M., Faustino A.M.R., Moreira R., Mela P., Loureiro L., Silva I., Gama M. (2015). A Novel Small-Caliber Bacterial Cellulose Vascular Prosthesis: Production, Characterization, and Preliminary In Vivo Testing. Macromol. Biosci..

[62] Pour H.S.-R., Khajavi R., Yazdanshenas M.E., Zahedi P., Mirjalili M. (2018). Cellulose acetate/poly(vinyl alcohol) hybrid fibrous mat containing tetracycline hydrochloride and phenytoin sodium: Morphology, drug release, antibacterial, and cell culture studies. J. Bioact. Compat. Polym..

[63] Han Y., Li C., Cai Q., Bao X., Tang L., Ao H., Liu J., Jin M., Zhou Y., Wan Y. (2020). Studies on Bacterial Cellulose/Poly(Vinyl Alcohol) Hydrogel Composites as Tissue-Engineered Corneal Stroma. Biomed. Mater..

[64] Hu Y., Catchmark J. (2011). Integration of cellulases into bacterial cellulose: Toward bioabsorbable cellulose compo-sites. J. Biomed. Mater. Res. Part B Appl. Biomater..

[65] Inoue B.S., Streit S., Schneider A.L.D.S., Meier M.M. (2020). Bioactive bacterial cellulose membrane with prolonged release of chlorhexidine for dental medical application. Int. J. Biol. Macromol..

[66] Dutta S., Patel D., Lim K. (2019). Functional Cellulose-Based Hydrogels as Extracellular Matrices for Tissue Engineering. J. Biol. Eng..

[67] Hu Y., Catchmark J.M., Zhu Y., Abidi N., Zhou X., Wang J., Liang N. (2014). Engineering of porous bacterial cellulose toward human fibroblasts ingrowth for tissue engineering. J. Mater. Res..

[68] Torgbo S., Sukyai P. (2019). Fabrication of Microporous Bacterial Cellulose Embedded with Magnetite and Hydroxyap-atite Nanocomposite Scaffold For Bone Tissue Engineering. Mater. Chem. Phys..

[69] Wu J., Yin N., Chen S., Weibel D.B., Wang H. (2019). Simultaneous 3D cell distribution and bioactivity enhancement of bacterial cellulose (BC) scaffold for articular cartilage tissue engineering. Cellulose.

[70] Pang M., Huang Y., Meng F., Zhuang Y., Liu H., Du M., Ma Q., Wang Q., Chen Z., Chen L. (2020). Application of bacterial cellulose in skin and bone tissue engineering. Eur. Polym. J..

[71] Qiao H., Guo T., Zheng Y., Zhao L., Sun Y., Liu Y., Xie Y. (2018). A Novel Microporous Oxidized Bacterial Cellu-lose/Arginine Composite and Its Effect on Behavior of Fibroblast/Endothelial Cell. Carbohydr. Polym..

[72] Vasconcelos N.F., Andrade F.K., Vieira L.D.A.P., Vieira R.S., Vaz J.M., Chevallier P., Mantovani D., Borges M.D.F., Rosa M.D.F. (2020). Oxidized bacterial cellulose membrane as support for enzyme immobilization: Properties and morphological features. Cellulose.

[73] Mandour Y., Mohammed S., Menem M. (2019). Bacterial cellulose graft versus fat graft in closure of tympanic mem-brane perforation. Am. J. Otolaryngol..

[74] Kim J., Kim S.W., Park S., Lim K.-T., Seonwoo H., Kim Y., Hong B.H., Choung Y.-H., Chung J.H. (2013). Bacterial Cellulose Nanofibrillar Patch as a Wound Healing Platform of Tympanic Membrane Perforation. Adv. Healthc. Mater..

[75] Rebelo A., Liu C., Schäfer K., Saumer M., Yang G., Liu Y. (2019). Poly(4-Vinylaniline)/Polyaniline Bi-layer-Functionalized Bacterial Cellulose for Flexible Electrochemical Biosensors. Langmuir.

[76] Li D., Wei Q., Wang Q., Lv P., Wei Q. (2016). Preparation of Pd/Bacterial Cellulose Hybrid Nanofibers for Dopamine Detection. Molecules.

[77] Yu S., Ding L., Lin H., Wu W., Huang J. (2019). A novel optical fiber glucose biosensor based on carbon quantum dots-glucose oxidase/cellulose acetate complex sensitive film. Biosens. Bioelectron..

[78] Rao A., Sathiavelu A., Mythili S. (2017). Mini review on nanoimmobilization of lipase and cellulase for biofuel production. Biofuels.

[79] Galdino C.J.S., Maia A.D., Meira H.M., Souza T.C., Amorim J.D., Almeida F.C., Costa A.F., Sarubbo L.A. (2020). Use of a bacterial cellulose filter for the removal of oil from wastewater. Process. Biochem..

[80] Pirsa S., Shamusi T., Kia E.M. (2018). Smart films based on bacterial cellulose nanofibers modified by conductive polypyrrole and zinc oxide nanoparticles. J. Appl. Polym. Sci..

[81] Lizundia E., Rincón-Iglesias M., Lanceros-Méndez S. (2020). Combining cobalt ferrite and graphite with cellulose nanocrystals for magnetically active and electrically conducting mesoporous nanohybrids. Carbohydr. Polym..

[82] Chen X., Yuan F., Zhang H., Huang Y., Yang J., Sun D. (2016). Recent approaches and future prospects of bacterial cellulose-based electroconductive materials. J. Mater. Sci..

[83] Lay M., González I., Tarrés J.A., Pellicer N., Bun K.N., Vilaseca F. (2017). High electrical and electro-chemical properties in bacterial cellulose/polypyrrole membranes. Eur. Polym. J..

[84] Dutta H., Paul S.K. (2019). Kombucha Drink: Production, Quality, and Safety Aspects. Production and Management of Beverages.

[85] Dourado F., Gama M., Rodrigues A. (2017). A Review on The Toxicology and Dietetic Role of Bacterial Cellu-lose. Toxicol. Rep..

[86] Zhai X., Lin D., Liu D., Yang X. (2018). Emulsions stabilized by nanofibers from bacterial cellulose: New potential food-grade Pickering emulsions. Food Res. Int..

[87] Amnuaikit T., Chusuit T., Raknam P., Boonme P. (2011). Effects of A Cellulose Mask Synthesized by A Bacterium on Facial Skin Characteristics and User Satisfaction. Med. Devices Evid. Res..

[88] Maculotti D., Dassenno D. (2016). Management of Peristomal Skin Complications with Negative Pressure Wound Therapy: A Case Study. Anat. Physiol..

[89] Yokouchi M., Kubo A. (2018). Maintenance of tight junction barrier integrity in cell turnover and skin diseases. Exp. Derm..

[90] Ganesan A., Shaikh F., Bradley W., Blyth D., Bennett D., Petfield J., Carson M., Wells J., Tribble D. (2019). Classifica-tion of Trauma-Associated Invasive Fungal Infections to Support Wound Treatment Decisions. Emerg. Infect. Dis..

[91] Ribeiro D.M.L., Júnior A.R.C., De Macedo G.H.R.V., Chagas V.L., Silva L.D.S., Cutrim B.D.S., Santos D.M., Soares B.L.L., Zagmignan A., Miranda R.d.C.M.D. (2019). Polysaccharide-Based Formulations for Healing of Skin-Related Wound Infections: Lessons from Animal Models and Clinical Trials. Biomolecules.

[92] Fisher M.C., Gurr S.J., Cuomo C.A., Blehert D.S., Jin H., Stukenbrock E.H., Stajich J.E., Kahmann R., Boone C., Denning D.W. (2020). Threats Posed by the Fungal Kingdom to Humans, Wildlife, and Agriculture. mBio.

[93] (2019). Ameriburn.Org. Burn Incidence Fact Sheet—American Burn Association. https://Ameriburn.Org/Who-We-Are/Media/Burn-Incidence-Fact-Sheet/.

[94] Picheth G.F., Pirich C.L., Sierakowski M.R., Woehl M.A., Sakakibara C.N., De Souza C.F., Martin A.A., Da Silva R., De Freitas R.A. (2017). Bacterial cellulose in biomedical applications: A review. Int. J. Biol. Macromol..

[95] Chu M., Gao H., Liu S., Wang L., Jia Y., Gao M., Wan M., Xu C., Ren L. (2018). Functionalization of composite bacterial cellulose with C60nanoparticles for wound dressing and cancer therapy. RSC Adv..

[96] Howell R.S., Gorenstein S., Gillette B.M., Digregorio J., Criscitelli T., Davitz M.S., Woods J.S., Acerra M., Brem H. (2018). A Framework to Assist Providers in the Management of Patients with Chronic, Nonhealing Wounds. Adv. Ski. Wound Care.

[97] Stumpf T.R., Yang X., Zhang J., Cao X. (2018). In situ and ex situ modifications of bacterial cellulose for applications in tissue engineering. Mater. Sci. Eng. C.

[98] Chiaoprakobkij N., Seetabhawang S., Sanchavanakit N., Phisalaphong M. (2019). Fabrication and characterization of novel bacterial cellulose/alginate/gelatin biocomposite film. J. Biomater. Sci. Polym. Ed..

[99] Kim H. (2018). Wound Dressing Materials: The Essentials. J. Wound Manag. Res..

[100] Sionkowska A., Mężykowska O., Piątek J. (2019). Bacterial nanocelullose in biomedical applications: A review. Polym. Int..

[101] Tamahkar E., Bakhshpour M., Denizli A. (2019). Molecularly imprinted composite bacterial cellulose nanofibers for antibiotic release. J. Biomater. Sci. Polym. Ed..

[102] Weyell P., Beekmann U., Küpper C., Dederichs M., Thamm J., Fischer D., Kralisch D. (2019). Tailor-made material characteristics of bacterial cellulose for drug delivery applications in dentistry. Carbohydr. Polym..

[103] Hajmohammadi K., Zabihi R.E., Akbarzadeh K., Parizad N. (2020). Using a combination therapy to combat scalp necrosis: A case report. J. Med. Case Rep..

[104] Schwarzkopf R. (2018). Do Different Wound Dressings after Total Joint Arthroplasty Make a Difference? Do Different Wound Dressings After Total Joint Arthroplasty Make A Difference?. Orthop. Surg. Sports Med..

[105] Delli Santi G., Borgognone A. (2019). The Use of Epiprotect^®^, An Advanced Wound Dressing, To Heal Paediatric Pa-tients With Burns: A Pilot Study. Burn. Open.

[106] Pooja R., Vadodaria K., Vidhya S. (2019). Synthesis of bacterial cellulose and herbal extract for the development of wound dressing. Mater. Today Proc..

[107] Kaviani A., Zebarjad S.M., Javadpour S., Ayatollahi M., Bazargan-Lari R. (2019). Fabrication and characterization of low-cost freeze-gelated chitosan/collagen/hydroxyapatite hydrogel nanocomposite scaffold. Int. J. Polym. Anal. Charact..

[108] Wichai S., Chuysinuan P., Chaiarwut S., Ekabutr P., Supaphol P. (2019). Development of Bacterial Cellu-lose/Alginate/Chitosan Composites Incorporating Copper (II) Sulfate As An Antibacterial Wound Dressing. J. Drug Deliv. Sci. Technol..

[109] Cielecka I., Szustak M., Kalinowska H., Gendaszewska-Darmach E., Ryngajłło M., Maniukiewicz W., Bielecki S. (2019). Glycerol-Plasticized Bacterial Nanocellulose-Based Composites with Enhanced Flexibility And Liquid Sorption Capaci-ty. Cellulose.

[110] Figueiredo A., Silva N., Barros-Timmons A., Almeida A., Silvestre A., Freire C. (2015). Antimicrobial Bacterial Cellu-lose Nanocomposites Prepared by In Situ Polymerization Of 2-Aminoethyl Methacrylate. Carbohydr. Polym..

[111] Nguyen T., Ruksakulpiwat C., Ruksakulpiwat Y. (2020). Effect of Cellulose Nanofibers from Cassava Pulp on Physi-cal Properties of Poly(Lactic Acid) Biocomposites. J. Thermoplast. Compos. Mater..

[112] Aydogdu M.O., Altun E., Ahmed J., Gunduz O., Edirisinghe M. (2019). Fiber Forming Capability of Binary and Ternary Compositions in the Polymer System: Bacterial Cellulose-Polycaprolactone-Polylactic Acid. Polymers.

[113] Sun Y., Meng C., Zheng Y., Xie Y., He W., Wang Y., Qiao K., Yue L. (2018). The Effects of Two Biocompatible Plas-ticizers on The Performance of Dry Bacterial Cellulose Membrane: A Comparative Study. Cellulose.

[114] Rubina M.S., Pigaleva M.A., Butenko I.E., Budnikov A.V., Naumkin A.V., Gromovykh T.I., Lutsenko S.V., Vasil’Kov A.Y. (2019). The interaction effect of bacterial cellulose with gold nanoparticles obtained by metal-vapor synthesis. Дoклады Академии наук.

[115] Jalili Tabaii M., Emtiazi G. (2018). Transparent Nontoxic Antibacterial Wound Dressing Based on Silver Nano Parti-cle/Bacterial Cellulose Nano Composite Synthesized in The Presence of Tripolyphosphate. J. Drug Deliv. Sci. Technol..

[116] Mohseni M., Shamloo A., Aghababaei Z., Vossoughi M., Moravvej H. (2016). Antimicrobial Wound Dressing Con-taining Silver Sulfadiazine with High Biocompatibility: In Vitro Study. Artif. Organs.

[117] Mathew S., Snigdha S., Mathew J., Krishnankutty R.E. (2018). Poly(vinyl alcohol): Montmorillonite: Boiled rice water (starch) blend film reinforced with silver nanoparticles; characterization and antibacterial properties. Appl. Clay Sci..

[118] Aboelnaga A., Elmasry M., Adly O., Elbadawy M., Abbas A., Abdelrahman I., Salah O., Steinvall I. (2018). Microbi-al Cellulose Dressing Compared with Silver Sulphadiazine for The Treatment Of Partial Thickness Burns: A Prospective, Randomised, Clinical Trial. Burns.

[119] Hosseini H., Zirakjou A., Goodarzi V., Mousavi S., Khonakdar H., Zamanlui S. (2020). Lightweight aerogels based on bacterial cellulose/silver nanoparticles/polyaniline with tuning morphology of polyaniline and application in soft tissue engi-neering. Int. J. Biol. Macromol..

[120] Ma B., Chaudhary J., Zhu J., Sun B., Chen C., Sun D. (2020). Construction of silver nanoparticles anchored in carbon-ized bacterial cellulose with enhanced antibacterial properties. Colloids Surf. A Physicochem. Eng. Asp..

[121] Goi Y., Fujisawa S., Saito T., Yamane K., Kuroda K., Isogai A. (2019). Dual Functions of TEMPO-Oxidized Cellulose Nanofibers in Oil-in-Water Emulsions: A Pickering Emulsifier and a Unique Dispersion Stabilizer. Langmuir.

[122] Khan S., Ul-Islam M., Khattak W.A., Ullah M.W., Park J.K. (2015). Bacterial cellulose-titanium dioxide nanocomposites: Nanostructural characteristics, antibacterial mechanism, and biocompatibility. Cellulose.

[123] Zywicka A., Fijałkowski K., Junka A., Grzesiak J., El Fray M. (2018). Modification of Bacterial Cellulose with Quater-nary Ammonium Compounds Based on Fatty Acids and Amino Acids and The Effect on Antimicrobial Activity. Biom-Acromolecules.

[124] Napavichayanun S., Ampawong S., Harnsilpong T., Angspatt A., Aramwit P. (2018). Inflammatory reaction, clinical efficacy, and safety of bacterial cellulose wound dressing containing silk sericin and polyhexamethylene biguanide for wound treatment. Arch. Derm. Res..

[125] Ye S., Jiang L., Wu J., Su C., Huang C., Liu X., Shao W. (2018). Flexible Amoxicillin-Grafted Bacterial Cellulose Sponges for Wound Dressing: In Vitro and in Vivo Evaluation. ACS Appl. Mater. Interfaces.

[126] Wijaya C.J., Saputra S.N., Soetaredjo F.E., Putro J.N., Lin C.X., Kurniawan A., Ju Y.-H., Ismadji S. (2017). Cellulose nanocrystals from passion fruit peels waste as antibiotic drug carrier. Carbohydr. Polym..

[127] Yaşayan G., Karaca G., Akgüner Z.P., Öztürk A.B. (2020). Chitosan/collagen composite films as wound dressings encapsulating allantoin and lidocaine hydrochloride. Int. J. Polym. Mater..

[128] Beekmann U., Schmölz L., Lorkowski S., Werz O., Thamm J., Fischer D., Kralisch D. (2020). Process control and scale-up of modified bacterial cellulose production for tailor-made anti-inflammatory drug delivery systems. Carbohydr. Polym..

[129] Wahid F., Hu X.-H., Chu L., Jia S.-R., Xie Y.-Y., Zhong C. (2019). Development of bacterial cellulose/chitosan based semi-interpenetrating hydrogels with improved mechanical and antibacterial properties. Int. J. Biol. Macromol..

[130] Müller A., Wesarg F., Hessler N., Müller F., Kralisch D., Fischer D. (2014). Loading of Bacterial Nanocellulose Hy-drogels With Proteins Using A High-Speed Technique. Carbohydr. Polym..

[131] Fürsatz M., Skog M., Sivlér P., Palm E., Aronsson C., Skallberg A., Greczynski G., Khalaf H., Bengtsson T., Aili D. (2017). Functionalization of bacterial cellulose wound dressings with the antimicrobial peptide ε-poly-L-Lysine. Biomed. Mater..

[132] Yang G., Jiang H., Zheng W., Gong N., Chen L., Jiang X., Yanga G. (2019). Correction: Bacterial cellulose–hyaluronan nanocomposite biomaterials as wound dressings for severe skin injury repair. J. Mater. Chem. B.

[133] Fallacara A., Durini E., Vertuani S., Manfredini S. (2017). Hyaluronic Acid Fillers in Soft Tissue Regeneration. Facial Plast. Surg..

[134] (2020). 360researchreports.com. Global Microbial and Bacterial Cellulose Market—Industry Reports. https://www.360researchreports.com/global-microbial-and-bacterial-cellulose-market-15085121.

[135] Portela R., Leal C., Almeida P., Sobral R. (2019). Bacterial Cellulose: A Versatile Biopolymer for Wound Dressing Ap-plications. Microb. Biotechnol..

[136] Leppiniemi J., Lahtinen P., Paajanen A., Mahlberg R., Metsä-Kortelainen S., Pinormaa T., Pajari H., Vikholm-Lundin I., Pursula P., Blazevic V. (2017). 3D-Printable Bioactivated Nanocellulose–Alginate Hydrogels. ACS Appl. Mater. Interfaces.

[137] Núñez-Carmona E., Bertuna A., Abbatangelo M., Sberveglieri V., Comini E., Sberveglieri G. (2019). BC-MOS: The novel bacterial cellulose based MOS gas sensors. Mater. Lett..

[138] Vuorinen T., Laurila M.-M., Mangayil R., Karp M., Mäntysalo M. (2018). High Resolution E-Jet Printed Temperature Sensor on Artificial Skin. XXVI Brazilian Congress on Biomedical Engineering.

[139] Golubnitschaja O., Veeser L.S., Avishai E., Costigliola V. (2019). Wound Healing: Proof-of-Principle Model for the Modern Hospital: Patient Stratification, Prediction, Prevention and Personalisation of Treatment. The Modern Hospital.

[140] Badshah M., Ullah H., Wahid F., Khan T. (2020). Bacterial Cellulose Based Metallic Green Nanocomposites for Bio-medical and Pharmaceutical Applications. Curr. Pharm. Des..

[141] Eslahi N., Mahmoodi A., Mahmoudi N., Zandi N., Simchi A. (2019). Processing and Properties of Nanofibrous Bacte-rial Cellulose-Containing Polymer Composites: A Review of Recent Advances for Biomedical Applications. Polym. Rev..

[142] Gorgieva S., Trcek J. (2019). Bacterial Cellulose: Production, Modification and Perspectives in Biomedical Applications. Nanomaterials.

[143] De Oliveira Barud H.G., da Silva R.R., da Silva Barud H., Tercjak A., Gutierrez J., Lustri W.R., de Oliveira O.B., Ri-beiro S.J.L. (2016). A multipurpose natural and renewable polymer in medical applications: Bacterial cellulose. Carbohydr. Polym..

[144] Rjwade J.M., Paknikar K.M., Kumbhar J.V. (2015). Applications of bacterial cellulose and its composites in biomedi-cine. Appl. Microbiol. Biotechnol..

[145] Hasan N., Rahman L., Kim S.-H., Cao J., Arjuna A., Lallo S., Jhun B.H., Yoo J.-W. (2020). Recent advances of nanocellulose in drug delivery systems. J. Pharm. Investig..

[146] Mohite B.V., Patil S.V. (2014). A novel biomaterial: Bacterial cellulose and its new era applications. Biotechnol. Appl. Biochem..

